# Comparing the number and length of primary care consultations in people with and without intellectual disabilities and health needs: observational cohort study using electronic health records

**DOI:** 10.1093/fampra/cmac135

**Published:** 2022-11-28

**Authors:** Freya Tyrer, Richard Morriss, Reza Kiani, Satheesh K Gangadharan, Harish Kundaje, Mark J Rutherford

**Affiliations:** Department of Population Health Sciences, University of Leicester, Leicester, United Kingdom; Institute of Mental Health, University of Nottingham, Nottingham, United Kingdom; Department of Population Health Sciences, University of Leicester, Leicester, United Kingdom; Leicestershire Learning Disability Services (Psychiatry), Leicestershire Partnership NHS Trust, Leicester, United Kingdom; Department of Population Health Sciences, University of Leicester, Leicester, United Kingdom; Leicestershire Learning Disability Services (Psychiatry), Leicestershire Partnership NHS Trust, Leicester, United Kingdom; Lakeside Healthcare, NHS General Practice, Corby, United Kingdom; Department of Population Health Sciences, University of Leicester, Leicester, United Kingdom

**Keywords:** GP consultations, health needs, intellectual disability, primary care

## Abstract

**Background:**

In the United Kingdom, 15-min appointments with the general practitioner (GP) are recommended for people with complex health conditions, including intellectual disabilities and health needs, but we do not know whether this happens.

**Aims:**

We compared number and length of primary care consultations (GP, nurse, other allied health, other) for people with and without intellectual disabilities and health needs.

**Methods:**

Linked primary care data from the Clinical Practice Research Datalink (CPRD) in England were used to investigate face-to-face and telephone primary care consultations in 2017–2019. Health needs investigated were: epilepsy; incontinence; severe visual/hearing impairments; severe mobility difficulties; cerebral palsy; and percutaneous endoscopic gastrostomy feeding. Age and gender-standardized consultation rates per year (Poisson), duration of consultations, and the proportion of “long consultations” (≥15 min) were reported.

**Results:**

People with intellectual disabilities (*n* = 7,794) had 1.9 times as many GP consultations per year as those without (*n* = 176,807; consultation rate ratio = 1.87 [95% confidence interval 1.86–1.89]). Consultation rates with nurses and allied healthcare professionals were also twice as high. Mean GP consultation time was 9–10 min regardless of intellectual disability/health need status. Long GP consultations were less common in people with intellectual disabilities (18.2% [17.8–18.7] vs. 20.9% [20.8–21.0]). Long consultations with practice nurses were more common in people with health needs, particularly severe visual loss.

**Conclusions:**

People with intellectual disabilities and/or health needs tend to have more, rather than longer, GP consultations compared with the rest of the population. We recommend further investigation into the role of practice nurses to support people with intellectual disabilities and health needs.

**Key messages**

- Intellectual disabilities and health needs are often managed in primary care.
- People with intellectual disabilities have more primary care consultations.
- GP consultations are no longer for people with intellectual disabilities.
- GP consultations are no longer for people with health needs.
- Practice nurse consultations are longer for people with health needs.

## Introduction

Addressing the burden of health inequalities is now a global priority.^1^ People with intellectual disabilities are a priority group to target because they are more likely than the general population to be affected by complex health needs, including epilepsy and sensory impairments.^2^ Whilst there are measures in place to reduce health inequalities in this population such as annual health checks,^3,4^ mortality data suggest that the situation has not improved, despite some deaths being potentially avoidable.^5^

As primary care is the first point of contact for most people with intellectual disabilities, general practitioners (GPs) and other primary care healthcare professionals have a crucial role to play in the implementation of strategies to reduce health inequalities. In England, where this study is set, primary care initiatives have included incentives to maintain intellectual disability registers (2006) and annual health checks (2008, 2014) to improve information sharing and identification of unmet health needs.^3,4,6^ Under the Equality Act 2010^7^ and Disability Discrimination Act 2005,^8^ GP surgeries are expected to make reasonable adjustments for people with intellectual disabilities, including longer appointments if necessary.^9^ However, management of long-term conditions in primary care has been found to be poor in people with intellectual disabilities^10^ and there are more emergency admissions that could be avoided with high-quality primary care.^11^ GPs have also reported lack of support in making clinical decisions for people with intellectual disabilities.^12^

In the United Kingdom, in 2016, recommendations were put forward by the British Medical Association (BMA) to increase appointment times to 15 min for individuals with complex health conditions.^13^ It seems likely that these individuals will include a significant proportion of people with intellectual disabilities owing to their high prevalence of complex health conditions.^2^ However, this has not yet been investigated. Previous UK research suggests that overall GP consultation times may be longer for people with multiple long-term health conditions^14^ but there is no evidence to suggest that they are longer for people with intellectual disabilities.^15^ In fact, it is arguable whether it is feasible to increase consultation times at all given that workload pressures are very high among an already depleting GP workforce.^16,17^ Moreover, the demand for primary care services is likely to increase in the future owing to a growing ageing population and rise in the prevalence of multiple long-term conditions.^18,19^ This and cuts to funding^20^ have led to GPs delegating some tasks to practice nurses, allied health professionals, or nonclinical staff in order to manage their workload more effectively.^21^ Therefore, other health professionals involved in primary care could have an important role to play. To our knowledge, this has not yet been investigated.

The aim of this study was to assess whether primary care consultation rates and length of consultations are higher for people with intellectual disabilities and/or health needs compared with the rest of the population. Given BMA guidance to increase GP consultation times to 15 min,^13^ we have focussed on GP consultations. However, we also aimed to investigate consultations with practice nurses and allied health professionals to determine their role in the management of intellectual disabilities and health needs.

## Methods

### Data sources

This study followed the Reporting of studies Conducted using Observational Routinely-collected health Data (RECORD) checklist^22^ (see Supplementary Table S1). We used the Clinical Practice Research Datalink (CPRD GOLD), linked (person-level) with hospital episode statistics (HES) and death registrations from the Office for National Statistics (approved study protocol number: 19_267). The CPRD is an electronic health record primary care research database, which is broadly representative of the national population in terms of age, gender, and ethnicity.^23^ Only GP surgeries in England consenting to linkage with HES and deaths data (approximately 75% of CPRD surgeries in England) were included in this study. The study population was drawn from a sample of individuals aged 10 years old and over with intellectual disabilities and a random sample of individuals without intellectual disabilities (also 10 years and over) between 2000 and 2019 (see data flow diagram; Supplementary Fig. S1). For this work, the study population was restricted to individuals in the 2017–2019 cohort period. This study population has been described previously^24^ and comprised 7,794 individuals with intellectual disabilities and a random sample of 176,807 people without intellectual disabilities between 2017 and 2019 (see data flow diagram; Supplementary Fig. S1).

### Intellectual disabilities, health needs, and consultations

Diagnostic codes are reported in the supplementary material (Supplementary Table S2). Choice of health needs was based on the literature^15^ and discussions with people with intellectual disabilities and family members as being sufficiently severe and life-long to impact on health and quality of life for those individuals affected. They were: epilepsy; incontinence; severe visual loss; severe hearing impairment; feeding via a percutaneous endoscopic gastrostomy (PEG) tube, severe mobility difficulties; and cerebral palsy, occurring within 10 years of cohort entry (with the exception of cerebral palsy which was considered life-long). As well as individual health needs, people with none of the health needs, 1 or more health need, and 2 or more health needs at baseline were investigated to assess any dose–response effects.

The methods for handling primary care consultations were based on previous CPRD research.^25,26^ All consultations were measured as direct contact (face-to-face/telephone) in minutes with the patient—see Supplementary Table S4 for details on the categorization of face-to-face or telephone conversations. The role of the healthcare professional was categorized into: GP (senior partner, partner, assistant, associate, locum, registrar, sole practitioner, retainer); practice nurse; allied health/social care professional; and other—see Supplementary Table S3. Duration times (minutes that the patient’s record was opened for a consultation) on the same day were assumed to be part of the same consultation, with times of 0 and >60 min set to 0.5 and 60 min, respectively.

### Statistical methods

Date of entry into the cohort was defined as the latest date according to the person and GP surgery’s characteristics: 1 January 2017; date of GP registration; up-to-standard date (the CPRD’s quality indicator for the data held at the surgery); or date the individual turned 10 years old (to align with the eligibility criteria). To correctly allocate risk times and events, intellectual disability and health needs status were allowed to change during the observation window. Date of exit was defined as: date of last CPRD update (29 September 2019); date of death; date of last surgery update; or date of transfer out of the surgery, whichever was first.

Rate of primary care consultations per year was calculated using Poisson regression, allowing for differences in lengths of exposure. As consultations rates and duration are known to be associated with age and gender,^25^ 10-year-age and gender-standardized consultation rate ratios were calculated for people with intellectual disabilities (i.e. observed consultations) using age and gender-specific consultation rates in the comparison group (i.e. expected consultations) as the reference standard. Mean duration of consultations (in minutes) and exact confidence intervals were calculated. The age (10-year) and gender-standardized percentage of consultations ≥15 min, defined as “long consultations,” were also compared between groups. We also plotted the percentage of long consultations by GP surgery to assess variability in practice.

We conducted 2 sensitivity analyses to test the robustness of the findings: (i) we repeated the analyses restricted to individuals in the cohort for ≥6 months; and (ii) we excluded consultations that had been amended/truncated (i.e. 0 and ≥60 min).

## Results

Table 1 shows the characteristics of the study population over the observation period. Compared with the comparison group, people with intellectual disabilities were generally younger (median age 33.0 vs. 43.0 years) and there were more males (57.1% vs. 49.0%). The most common aetiological condition for intellectual disability was Down syndrome (10.9%). All of the health needs were substantially more prevalent among people with intellectual disabilities; the most common health need was epilepsy, which was present in 18.7% of individuals at baseline (compared with 1.1% in the comparison group).

**Table 1.** Baseline and follow-up characteristics of the study population by intellectual disability and health need status.

Characteristic	Intellectual disability		No intellectual disability^a^	
	Number/median	Percent/range	Number/median	Percent/range
Total	7,794	100.0	176,807	100.0
Demographic characteristics				
Age (years)	33.0	10–101	43.0	10–108
Gender				
Male	4,448	57.1	86,669	49.0
Female	3,346	42.9	90,138	51.0
Ethnicity				
White	6,002	77.0	119,403	67.5
South Asian	211	2.7	7,662	4.3
Black	207	2.7	6,236	3.5
Other	280	3.6	8,907	5.0
Not known	1,094	14.0	34,599	19.6
Observation period				
Length in cohort (years)	1.5	>0.0–2.7	1.9	>0.0–2.7
Most common genetic/chromosomal syndromes^b^				
Down syndrome	848	10.9	—	
Fragile X syndrome	151	1.9	—	
Tuberous sclerosis	60	0.8	—	
Edward syndrome	29	0.4	—	
Prader–Willi syndrome	27	0.3	—	
Health needs				
None (at baseline or follow-up)	4,174	53.6	159,716	90.3
Epilepsy				
Baseline	1,456	18.7	2,004	1.1
During follow-up	55	0.7	201	0.1
Incontinence				
Baseline	1,039	13.3	6,649	3.8
During follow-up	214	2.7	1,177	0.7
Severe visual loss				
Baseline	1,015	13.0	1,075	0.6
During follow-up	227	2.9	204	0.1
Severe hearing impairment				
Baseline	551	7.1	5,253	3.0
During follow-up	67	0.9	592	0.3
Severe mobility difficulties				
Baseline	818	10.5	1,280	0.7
During follow-up	174	2.2	570	0.3
Cerebral palsy				
Baseline	658	8.4	261	0.1
During follow-up	20	0.3	6	<0.1
PEG^c^ feeding				
Baseline	132	1.7	180	0.1
During follow-up	20	0.3	54	<0.1

^a^ *n* = 440 individuals moved from no intellectual disability to intellectual disability sample at first diagnosis during observation window.

^b^ *n* = 831 (10.7%) with phenylketonuria (not defined as a specific syndrome for this study).

^c^Percutaneous endoscopic gastrostomy.

**Fig. 1.**
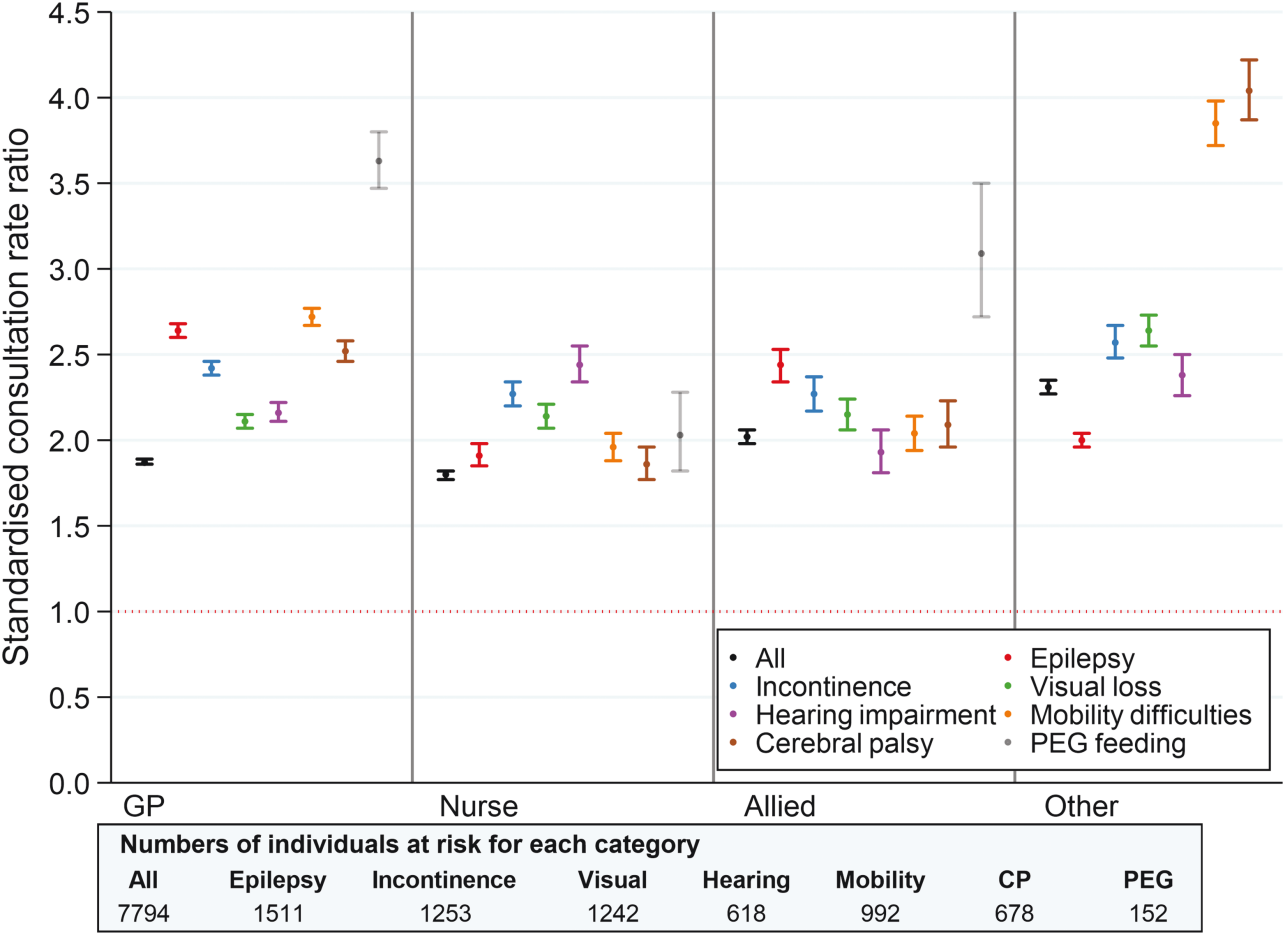
Standardized consultation rate ratio by health professional role and individual health needs (standardized by the age [10-year] and gender distribution in the entire population without intellectual disabilities; “other” category not shown for PEG feeding owing to small numbers).

In total, there were 96,244 face-to-face/telephone consultations in people with intellectual disabilities and 1,579,364 consultations in people without intellectual disabilities during 2017–2019. Figure 1 shows the standardized consultation rate ratio for people with intellectual disabilities by individual health needs and role of health professional (consultation rates are shown in Supplementary Table S5).

Overall, people with intellectual disabilities had 1.9 times as many GP consultations as those without (consultation rate ratio = 1.87; [95% confidence interval: 1.86–1.89]), 1.8 times as many practice nurse consultations (ratio = 1.80 [1.77–1.82]) and twice as many allied health professional consultations (consultation rate ratio = 2.02 [1.98–2.66]). Consultation rates were higher in people with co-occurring health needs, in particular for PEG feeding (GP and allied health professional: ratio = 3.63 [3.47–3.80] and 3.09 [2.72–3.50], respectively). GP consultations were also higher in people with epilepsy and severe mobility difficulties (rate = 2.64 [2.60–2.68] and 2.72 [2.67–2.77], respectively).

**Fig. 2.**
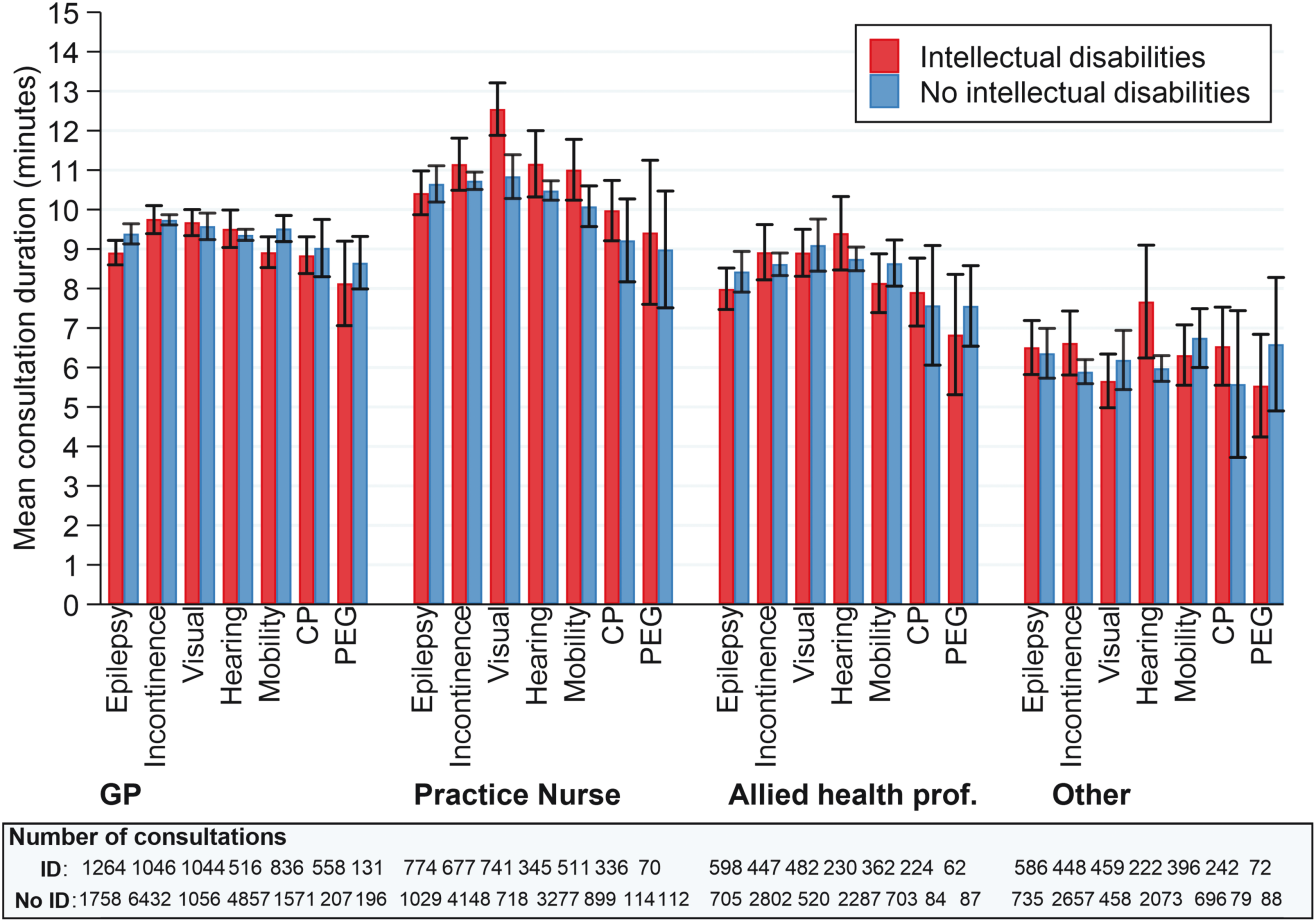
Mean duration of consultations by primary care role and individual health need status.

The mean duration of the GP consultations was 9.3 min (9.12–9.42) for people with intellectual disabilities and 9.4 min (9.38–9.44) in the comparison group (Supplementary Table S6). The average duration of GP consultations was 9–10 min for people with and without intellectual disability and/or health needs. Consultations with practice nurses were generally longer (intellectual disability vs. no intellectual disability: mean duration 10.8 vs. 10.3 min) and consultations with allied health professionals were generally shorter (8.8 vs. 8.7 min, respectively). The mean duration of GP consultations was similar across health needs and did not appear to be different by intellectual disability status (Fig. 2). For practice nurses, consultations were longer for people with health needs who also had intellectual disabilities, most notably severe visual loss (12.6 min [11.9–13.2] vs. 10.8 min [10.3–11.4]).

The percentage of consultations (standardized by age and gender) that were ≥15 min (i.e. long consultations) for both groups by health professional role and individual health need are shown in Table 2. People with intellectual disabilities generally had a lower proportion of long consultations with the GP compared with those without (18.2% [17.8–18.9] vs. 20.9% [20.8–21.0]). This was fairly consistent across all of the health needs under investigation.

**Table 2.** The percentage of “long consultations” (≥15 min) in people with and without intellectual disabilities by health need status and role of health professional^a^^,^^b^.

Health need	Number of individuals	Percentage of “long consultations”		Number of individuals	Percentage of “long consultations”	
GP consultations						
All	5,809	18.22	(17.79–18.66)	119,607	20.86	(20.79–20.95)
Number of health needs						
None	3,084	18.46	(17.65–19.28)	107,489	20.69	(20.60–20.78)
1+	2,725	18.11	(17.59–18.63)	12,118	21.86	(21.62–22.09)
2+	1,203	18.25	(17.47–19.02)	1,240	21.58	(20.82–22.33)
Individual health needs						
Epilepsy	1,264	16.00	(15.26–16.75)	1,758	21.50	(20.93–22.06)
Incontinence	1,046	19.94	(19.16–20.71)	6,432	22.61	(22.27–22.94)
Severe visual loss	1,044	19.73	(18.77–20.70)	1,056	22.25	(21.24–23.25)
Severe hearing impairment	516	20.72	(19.61–21.82)	4,857	21.67	(21.21–22.13)
Severe mobility difficulties	836	17.13	(16.32–17.94)	1,571	20.12	(19.41–20.83)
Cerebral palsy	558	13.76	(12.79–14.74)	207	19.19	(16.95–21.43)
PEG feeding	131	12.69	(10.71–14.67)	196	21.52	(19.75–23.29)
Practice nurse consultations						
All	3,459	26.53	(25.47–27.58)	74,585	26.39	(26.23–26.55)
Number of health needs						
None	1,714	24.45	(22.71–26.19)	66,554	26.21	(26.03–26.38)
1+	1,745	27.73	(26.30–29.17)	18,031	27.64	(27.16–28.13)
2+	797	32.00	(30.18–33.83)	795	29.14	(27.13–31.15)
Individual health needs						
Epilepsy	774	25.35	(23.15–27.55)	1,029	26.80	(25.54–28.05)
Incontinence	677	28.43	(26.77–30.09)	4,148	28.04	(27.35–28.73)
Severe visual loss	741	30.71	(28.24–33.19)	718	29.57	(27.63–31.51)
Severe hearing impairment	345	25.30	(23.19–27.40)	3,277	27.38	(26.46–28.29)
Severe mobility difficulties	511	28.88	(26.63–31.13)	899	29.58	(27.84–31.32)
Cerebral palsy	336	—^c^		114	—^c^	
PEG feeding	70	16.68	(12.14–21.21)	112	25.36	(21.94–28.78)
Allied health professional consultations						
All	2,483	19.68	(18.46–20.90)	45,903	19.53	(19.34–19.73)
Number of health needs						
None	1,266	21.20	(19.16–23.23)	40,334	19.43	(19.22–19.64)
1+	1,217	20.14	(17.87–22.40)	5,569	19.66	(19.10–20.22)
2+	535	21.83	(19.25–24.40)	564	20.94	(19.10–22.79)
Individual health needs						
Epilepsy	598	15.48	(13.71–17.24)	705	19.86	(18.46–21.27)
Incontinence	447	20.27	(18.12–22.42)	2,802	19.53	(18.74–20.32)
Severe visual loss	482	21.56	(18.80–24.32)	520	20.52	(18.44–22.59)
Severe hearing impairment	230	20.36	(17.73–22.99)	2,287	19.86	(18.79–20.93)
Severe mobility difficulties	362	19.31	(16.35–22.28)	703	23.09	(21.19–24.99)
Cerebral palsy	224	—^c^		84	—^c^	
PEG feeding	62	14.38	(8.93–19.84)	87	20.72	(16.31–25.14)
Other consultations						
All	2,308	10.22	(9.15–11.29)	42,269	8.24	(8.12–8.36)
Number of health needs						
None	1,091	8.82	(7.71–9.94)	37,128	8.18	(8.05–8.31)
1+	1,217	10.44	(9.18–11.69)	5,141	8.56	(8.23–8.90)
2+	565	10.72	(9.05–12.39)	574	8.99	(7.85–10.14)
Individual health needs						
Epilepsy	586	9.26	(8.05–10.46)	735	9.25	(8.36–10.15)
Incontinence	448	12.42	(10.78–14.06)	2,657	8.65	(8.18–9.13)
Severe visual loss	459	9.48	(7.78–11.18)	458	8.86	(6.94–10.77)
Severe hearing impairment	222	8.74	(7.14–10.33)	2,073	8.26	(7.64–8.89)
Severe mobility difficulties	396	10.42	(8.57–12.26)	696	10.55	(9.38–11.72)
Cerebral palsy	242	8.35	(6.68–10.01)	79	11.15	(7.74–14.57)
PEG feeding	72	8.71	(6.27–11.16)	88	8.95	(6.73–11.17)

Total number of GP consultations: *n* = 56,462 (intellectual disabilities); *n* = 934,577 (without intellectual disabilities); nurse consultations: *n* = 15,702 (intellectual disabilities); *n =* 278,897 (without intellectual disabilities); allied health professional consultations: *n* = 9,882 (intellectual disabilities); *n* = 161,908 (without intellectual disabilities); other professionals: *n* = 14,198 (intellectual disabilities); *n* = 203,982 (without intellectual disabilities).

^a^Individuals with 1+ consultation only.

^b^Standardized by 10-year-age group and gender.

^c^Standardized not possible owing to small numbers.

Practice nurses and allied health professionals had a similar proportion of long consultations with people with and without intellectual disabilities. However, people with health needs, regardless of intellectual disability status, had a greater proportion of long consultations with practice nurses (none vs. 2+ health needs; 24.5% [22.7–26.2] vs. 32.0% [30.2–33.8] for intellectual disabilities and 26.2% [26.0–26.4] vs. 29.1% [27.1–31.2] for no intellectual disabilities). For both groups, long consultations with practice nurses were more prevalent in people with severe visual loss (all individuals vs. severe visual loss: 30.7% [28.2–33.2] vs. 26.5% [25.5–27.6] for intellectual disabilities and 29.6% [27.6–31.5] vs. 26.4% [26.2–26.6] for no intellectual disabilities).

**Fig. 3.**
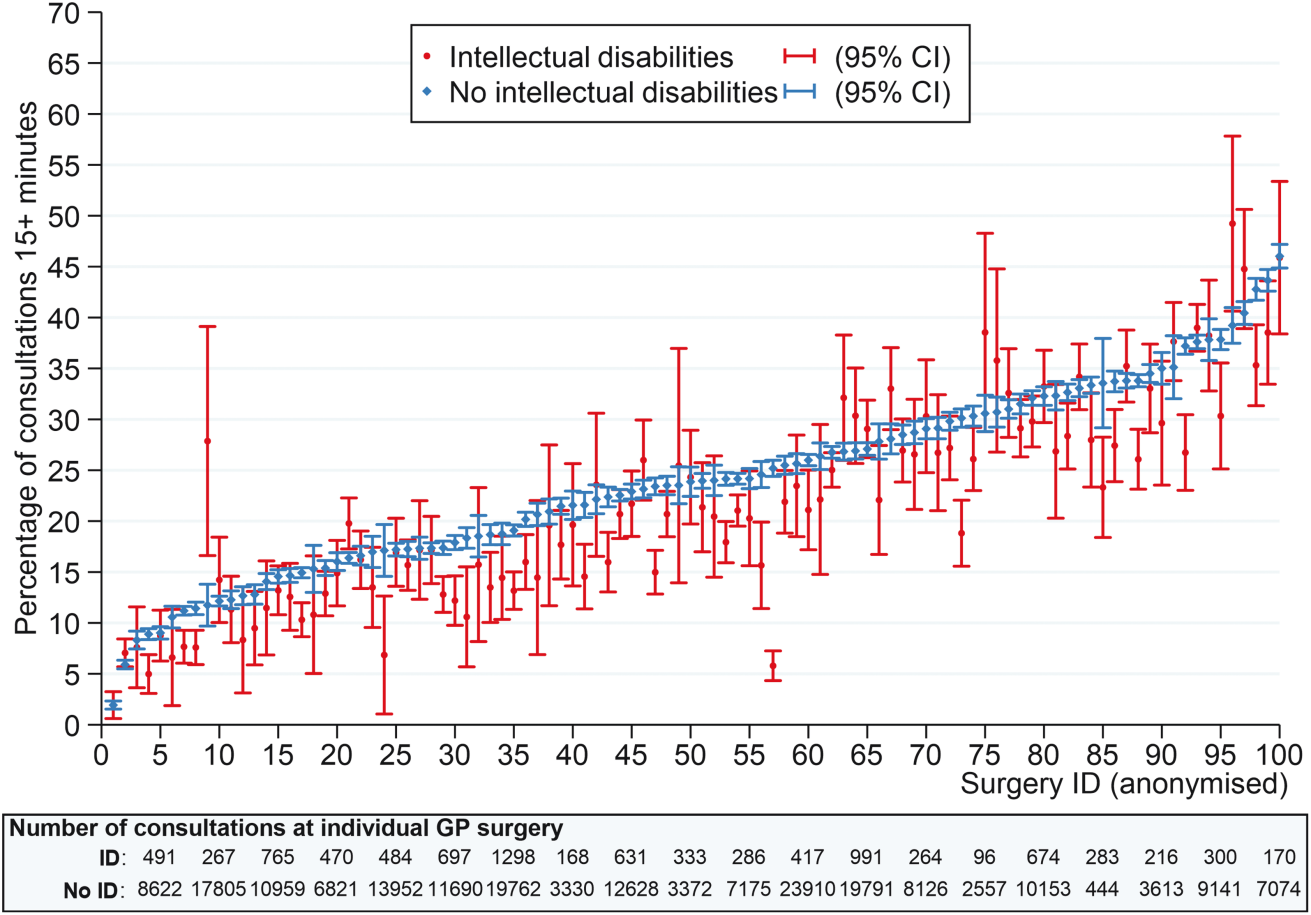
Percentage of “long consultations” (≥15 min) by GP surgery (100 GP surgeries in total; *n* = 25 surgeries were excluded with <50 GP consultations. Surgeries are ordered by the percentage of long consultations in the comparison cohort).

Figure 3 shows that there was wide variation in the percentage of long consultations by GP surgery, ranging from 2% (both groups) to 46% (both groups). There were no obvious differences in the proportion of long consultations by intellectual disability status by individual surgery, but it is notable from the figure that the proportion of long consultations appeared to be driven by the GP surgery rather than the individuals’ intellectual disability status (i.e. the blue and red markers followed the same trend).

The sensitivity analyses revealed similar overall findings when restricting to people in the cohort for ≥6 months (see Supplementary Tables S7 and S8). The exclusion of the very short (0.5 min) and very long GP consultations (those truncated to 60 min) lengthened the overall appointment times to closer to 10 min (Supplementary Table S8) but did not show discernible differences by intellectual disability or health need status.

## Discussion

In this study, people with intellectual disabilities had almost twice as many GP consultations as those without intellectual disabilities but their duration was about the same (9.3–9.4 min). There was also no evidence that long consultations (i.e. ≥15 min) with the GP were more common for people with intellectual disabilities and/or health needs than other individuals. However, long consultations with practice nurses were more common in people with health needs, regardless of intellectual disability status, particularly for severe visual loss where around 4% (percentage point difference) more consultations were ≥15 min compared with people without any health needs.

Our findings that people with intellectual disabilities had 1.8–1.9 as many GP/nurse consultations as people without intellectual disabilities largely correspond with previous research in the United Kingdom and Netherlands where number of consultations (including nurse consultations) was 1.6 and 1.7 times higher, respectively.^15,27^ The average duration of consultations was 9.3–9.4 min for this study, which is close to UK population estimates of 9.2 min.^25^ We also found that a marginally lower proportion of people with intellectual disabilities had long consultations (≥15 min) which corresponds with previous literature of >10 min consultations.^15^ We did not find any relationship between health needs and longer duration of appointments which differs from UK findings on presence and number of multiple long-term health conditions.^14^ This may reflect the nature of the health needs investigated as they do not typically require lifestyle advice or clinical measurement.

Overall, we found no evidence that long consultations with the GP were more common for people with intellectual disabilities and/or health needs than other individuals. However, we did find some evidence that long consultations with the practice nurse were more common in people with health needs irrespective of intellectual disability status. Practice nurses play a crucial role in the identification and management of individuals with long-term conditions as well as supporting those at risk of developing long-term conditions.^28^ We speculate that people with visual impairment may have had other co-occurring conditions for which specialist nursing support and lifestyle advice are necessary, such as diabetes,^29^ which may explain why these consultations were typically longer. With an ageing population and increasing demand on GPs, the role of nurses is becoming more important. Further work is needed to assess the efficacy, efficiency, and cost–benefit of the role of practice nurses to support people with complex health needs.

In this study, we found a large variability in the proportion of GP consultations that were “long.” There is known to be variability in consultation times by GP surgery,^30^ which is likely to be driven by a combination of the characteristics of the patients seen and workload pressures of the surgery. Although we standardized for age and gender, we did not investigate surgery-specific characteristics, such as training status, which is known to influence consultation rates and duration^25^ or urban/rural location. We also did not investigate the effect of additional patient-specific characteristics, such as ethnicity.

Another key consideration is that long GP consultations may not necessarily be a good thing. There is little evidence that individuals’ experiences are better if GP consultations are longer^31^ and efficiency and good communication (i.e. the quality of the consultation) are likely to be more important than length. The expressed rationale for introducing 15-min appointments for people with complex conditions was to improve decision making, case management, and efficiency by reducing the “administrative burden outside clinic times,” but also to ensure safe and high-quality patient care.^13^ Our data suggest that these recommendations have not been followed, and we recommend further qualitative and survey-based research to investigate why this is the case. It is also worth noting that, in this study, people with intellectual disabilities had more consultations with the GP which, effectively, equates to more consultation time—and also aligns with previous evidence that higher consultation rates are associated with shorter duration of appointments.^25^ Given the current workload pressures on GPs,^16,17^ this may be the only way in which all patients can be seen and needs met in a timely manner.

This study has the advantage of using a large, population-based sample of electronic primary care health records in England that has been extensively used for research purposes. However, we were unable to capture severity of intellectual disabilities or validate the diagnoses used. We were also not able to capture variability in the severity of health needs between the intellectual disability and general population or whether the health needs resolve over time. A particular concern is incontinence which may resolve during some life events, such as postpregnancy^32^ thereby affecting proportionally more women without intellectual disabilities because they are more likely to have children.^33^ However, this did not appear to influence the findings substantially as incontinence was not a significant indicator of consultation time or length. Another key limitation of this study is the metric used to record consultations. The length of opening the electronic health record for a face-to-face or telephone consultation may not have been the same as length of the appointment, for example, if the record were opened at the end of the consultation only.^25^ The exclusion of the very short (0.5 min) and very long GP consultations (those truncated to 60 min) lengthened the overall appointment times to closer to 10 min but did not show discernible differences by intellectual disability or health need status. The choice of 15 min for “long consultations” was pragmatic based on recommendations to increase appointment times to 15 min for complex health conditions^13^ but may not have been a suitable threshold. Further, this study took place before COVID-19. We speculate that telephone consultations with people with intellectual disabilities may be longer owing to communication difficulties or requirements to communicate in different ways. We are also not able to determine whether a carer was present at the consultations which is likely to have affected consultation times, particularly for telephone consultations.

Finally, we have found that GPs tend to translate reasonable adjustments for people with intellectual disabilities into more, rather than longer, consultations. This is likely to reflect GP demands to provide “same day access” to patients and it may not be feasible to lengthen appointment times. We recommend coordinated approaches between primary, secondary, and hospital care to optimize the consultation process for people with intellectual disabilities and/or complex health needs, also ensuring that reasonable adjustments are made. We also recommend an evaluation into the role of practice nurses to support people with intellectual disabilities and complex health needs.

## Supplementary material

Supplementary material is available at *Family Practice* online.

cmac135_suppl_Supplementary_Material

## Data Availability

Data for this study were obtained from the Clinical Practice Research Datalink (CPRD), provided by the UK MRHA. The authors’ licence for using these data does not allow sharing of raw data with third parties. Information about access to CPRD data is available here: https://www.cprd.com/research-applications. Researchers should contact the ISAC Secretariat at isac@cprd.com for further details.
