# Supplementary material for: Comparing the number and length of primary care consultations in people with and without intellectual disabilities and health needs: observational cohort study using electronic health records
Source: Fam Pract. 2022 Nov 28;41(4):501–9. doi: 10.1093/fampra/cmac135 (PMC11324320; doi:10.1093/fampra/cmac135)
Supplement: cmac135_suppl_Supplementary_Material [file cmac135_suppl_supplementary_material.docx]

**Comparing the number and length of primary care consultations in people with and without intellectual disabilities and health needs: observational cohort study using electronic health records**

**Supplementary material**

**Table S1. RECORD^a^ checklist (Reporting of studies conducted using observational routinely-collected data;** [**https://www.record-statement.org/checklist.php**](https://www.record-statement.org/checklist.php)**)**

|  | **Item No.** | **STROBE items** | **Location in original manuscript where items are reported** | **RECORD items** | **Location in manuscript where items are reported** |
| --- | --- | --- | --- | --- | --- |
| **Title and Abstract** | | | | | |
|  | 1 | (a) Indicate the study’s design with a commonly used term in the title or the abstract | 🗹 **Title**. | RECORD 1.1: The type of data used should be specified in the title or abstract. When possible, the name of the databases used should be included. | 🗹 **Title** and **Abstract**. |
|  |  | (b) Provide in the abstract an informative and balanced summary of what was done and what was found | 🗹 **Abstract**. | RECORD 1.2: If applicable, the geographic region and timeframe within which the study took place should be reported in the title or abstract. | 🗹 Calendar periods (2017–2019) and England listed in **Abstract**. |
|  |  |  |  | RECORD 1.3: If linkage between databases was conducted for the study, this should be clearly stated in the title or abstract | 🗹 Linked data mentioned in the **Abstract**. |
| **Introduction** | | | | | |
| Background rationale | 2 | Explain the scientific background and rationale for the investigation being reported | 🗹 **Introduction**. |  | |
| Objectives | 3 | State specific objectives, including any prespecified hypotheses | 🗹 **Introduction**. |  | |
| **Methods** | | | | | |
| Study Design | 4 | Present key elements of study design early in the paper | 🗹 see **Data sources**. |  | |
| Setting | 5 | Describe the setting, locations, and relevant dates, including  periods of recruitment, exposure, follow-up, and data collection | 🗹 See **Data sources** and **Statistical Methods**. |  | |
| Participants | 6 | (a) *Cohort study* - Give the eligibility criteria, and the sources and methods of selection of participants. Describe methods of follow-up  *Case-control study* - Give the eligibility criteria, and the sources and methods of case ascertainment and control selection. Give the rationale for the choice of cases and controls  *Cross-sectional study* - Give the eligibility criteria, and the sources and methods of selection of participants | 🗹 **Data sources**  Additional cohort entry and exit criteria are listed under **Statistical Methods**. | RECORD 6.1: The methods of study population selection (such as codes or algorithms used to identify subjects) should be listed in detail. If this is not possible, an explanation should be provided. | 🗹 All codes listed in the supplementary material (**Table S2**) & data flow diagram (**Figure S1**). |
|  |  | (b) *Cohort study* For matched studies, give matching criteria and number of exposed and unexposed  *Case-control study* - For matched studies, give matching criteria and the number of controls per case | N/A. | RECORD 6.2: Any validation studies of the codes or algorithms used to select the population should be referenced. If validation was conducted for this study and not published elsewhere, detailed methods and results should be provided. | 🗹 All codes listed in **Table S2**. Relevant literature cited in **Methods** (under heading ‘**Intellectual disabilities, health needs and consultations**’). |
|  |  |  |  | RECORD 6.3: If the study involved linkage of databases, consider use of a flow diagram or other graphical display to demonstrate the data linkage process, including the number of individuals with linked data at each stage. | 🗹 **Figure S1**. |
| Variables | 7 | Clearly define all outcomes, exposures, predictors, potential confounders, and effect modifiers. Give diagnostic criteria, if applicable. | 🗹 See **Methods** (under heading ‘**Intellectual disabilities, health needs and consultations**’). | RECORD 7.1: A complete list of codes and algorithms used to classify exposures, outcomes, confounders, and effect modifiers should be provided. If these cannot be reported, an explanation should be provided. | 🗹 **Table S2**. |
| Data sources/ measurement | 8 | For each variable of interest, give sources of data and details of methods of assessment (measurement).  Describe comparability of assessment methods if there is more than one group | 🗹 See **Methods** (under heading ‘**Intellectual disabilities, health needs and consultations**’). |  | |
| Bias | 9 | Describe any efforts to address potential sources of bias | 🗹 Errors in case ascertainment mitigated by excluding most recent diagnoses >10years from cohort entry (**Data sources**). Immortal time bias reduced by allowing intellectual disability status to change over time (**Statistical Methods**). |  | |
| Study size | 10 | Explain how the study size was arrived at | 🗷 All of the exposed population on the CPRD meeting eligibility criteria were selected. Feasibility counts for sample size are included in the cited ISAC protocol number. |  | |
| Quantitative variables | 11 | Explain how quantitative variables were handled in the analyses. If applicable, describe which groupings were chosen, and why | 🗹 See **Data Sources** & **Statistical Methods**. |  | |
| Statistical methods | 12 | (a) Describe all statistical methods, including those used to control for confounding | 🗹 See **Statistical Methods**. |  | |
|  |  | (b) Describe any methods used to examine subgroups and interactions | 🗹 See **Statistical Methods**. |  |  |
|  |  | (c) Explain how missing data were addressed | 🗷 N/A for this study. |  |  |
|  |  | (d) *Cohort study* - If applicable, explain how loss to follow-up was addressed  *Case-control study* - If applicable, explain how matching of cases and controls was addressed  *Cross-sectional study* - If applicable, describe analytical methods taking account of sampling strategy | 🗹 Right censoring strategy described in **Statistical Methods**. |  |  |
|  |  | (e) Describe any sensitivity analyses | 🗹 The sensitivity analyses conducted (**Statistical Methods**) and subsequent findings are presented in **Tables S7** and **S8**. |  |  |
| Data access and cleaning methods |  | | | RECORD 12.1: Authors should describe the extent to which the investigators had access to the database population used to create the study population. | 🗹 This is an established research database that has been quality assessed and checked for internal validity. |
|  |  |  |  | RECORD 12.2: Authors should provide information on the data cleaning methods used in the study. | 🗹 Relevant codes have been described for this study (**Table S2**). Data cleaning of consultations data cited in **Methods**. |
| Linkage |  | | | RECORD 12.3: State whether the study included person-level, institutional-level, or other data linkage across two or more databases. The methods of linkage and methods of linkage quality evaluation should be provided. | 🗹 Person-level linkage was used for this analysis as specified in the methods (**Data Sources**). |
| **Results** | | | | | |
| Participants | 13 | (a) Report the numbers of individuals at each stage of the study (*e.g.*, numbers potentially eligible, examined for eligibility, confirmed eligible, included in the study, completing follow-up, and analysed) | 🗹 **Figure S1**. | RECORD 13.1: Describe in detail the selection of the persons included in the study (*i.e.,* study population selection) including filtering based on data quality, data availability and linkage. The selection of included persons can be described in the text and/or by means of the study flow diagram. | 🗹 **Figure S1**. |
|  |  | (b) Give reasons for non- participation at each stage. | 🗹 **Figure S1**. |  |  |
|  |  | (c) Consider use of a flow diagram | 🗹 **Figure S1**. |  |  |
| Descriptive data | 14 | (a) Give characteristics of study participants (*e.g.*, demographic, clinical, social) and information on exposures and potential confounders | 🗹 **Table 1**. |  | |
|  |  | (b) Indicate the number of participants with missing data for each variable of interest | 🗹 Missing/unknown ethnicity listed in **Table 1**. |  |  |
|  |  | (c) *Cohort study* - summarise follow-up time (*e.g.*, average and total amount) | 🗹 **Table 1**. |  |  |
| Outcome data | 15 | *Cohort study* - Report numbers of outcome events or summary measures over time  *Case-control study* - Report numbers in each exposure category, or summary measures of exposure  *Cross-sectional study* - Report numbers of outcome events or summary measures | 🗹 **Table 1**. |  | |
|  | 16 | (a) Give unadjusted estimates and, if applicable, confounder- adjusted estimates and their precision (e.g., 95% confidence interval). Make clear which confounders were adjusted for and why they were included | 🗹 95% confidence intervals reported in the figures and supplementary tables. |  | |
|  |  | (b) Report category boundaries when continuous variables were categorized | N/A. |  |  |
|  |  | (c) If relevant, consider translating estimates of relative risk into absolute risk for a meaningful time period | N/A. |  |  |
| Other analyses | 17 | Report other analyses done— e.g., analyses of subgroups and interactions, and sensitivity analyses | See **Tables S7** and **S8** for sensitivity analyses. |  | |
| **Discussion** | | | | | |
| Key results | 18 | Summarise key results with  reference to study objectives | 🗹 See **Discussion**. |  | |
| Limitations | 19 | Discuss limitations of the study, taking into account sources of potential bias or imprecision.  Discuss both direction and magnitude of any potential bias | 🗹 See **Discussion**. | RECORD 19.1: Discuss the implications of using data that were not created or collected to answer the specific research question(s). Include discussion of misclassification bias, unmeasured confounding, missing data, and changing eligibility over time, as they pertain to the study being  reported. | 🗹 Limitations of the metric used to record consultations, inability to collect severity information or whether carers were present are discussed in the **Discussion**. Implications of reporting data pre-COVID are also discussed. |
| Interpretation | 20 | Give a cautious overall interpretation of results considering objectives, limitations, multiplicity of analyses, results from similar studies, and other relevant evidence | 🗹 See **Discussion**. |  | |
| Generalisability | 21 | Discuss the generalisability (external validity) of the study results | 🗹 See **Discussion**. |  | |
| **Other Information** | | | | | |
| Funding | 22 | Give the source of funding and the role of the funders for the present study and, if applicable, for the original study on which the present article is based | 🗹 See **Funding**. |  | |
| Accessibility of protocol, raw data, and programming  code |  | | | RECORD 22.1: Authors should provide information on how to access any supplemental information such as the study protocol, raw data, or programming code. | 🗹 The CPRD’s ISAC study protocol ID is listed and available to view on-line (**Data sources**). Raw data are not available and an explanation is given for this in the **Data availability** statement. |

Checklist is protected under Creative Commons Attribution ([CC BY](http://creativecommons.org/licenses/by/4.0/)) licence.

^a^ Reference: Benchimol EI, Smeeth L, Guttmann A, Harron K, Moher D, Petersen I, Sørensen HT, von Elm E, Langan SM, the RECORD Working Committee. The REporting of studies Conducted using Observational Routinely-collected health Data (RECORD) Statement. *PLoS Medicine* 2015; 2(10): e1001885. https://doi.org/10.1371/journal.pmed.1001885

**Figure S1: Data flow diagram of individuals included in the study population from original extracted data^a^**

**
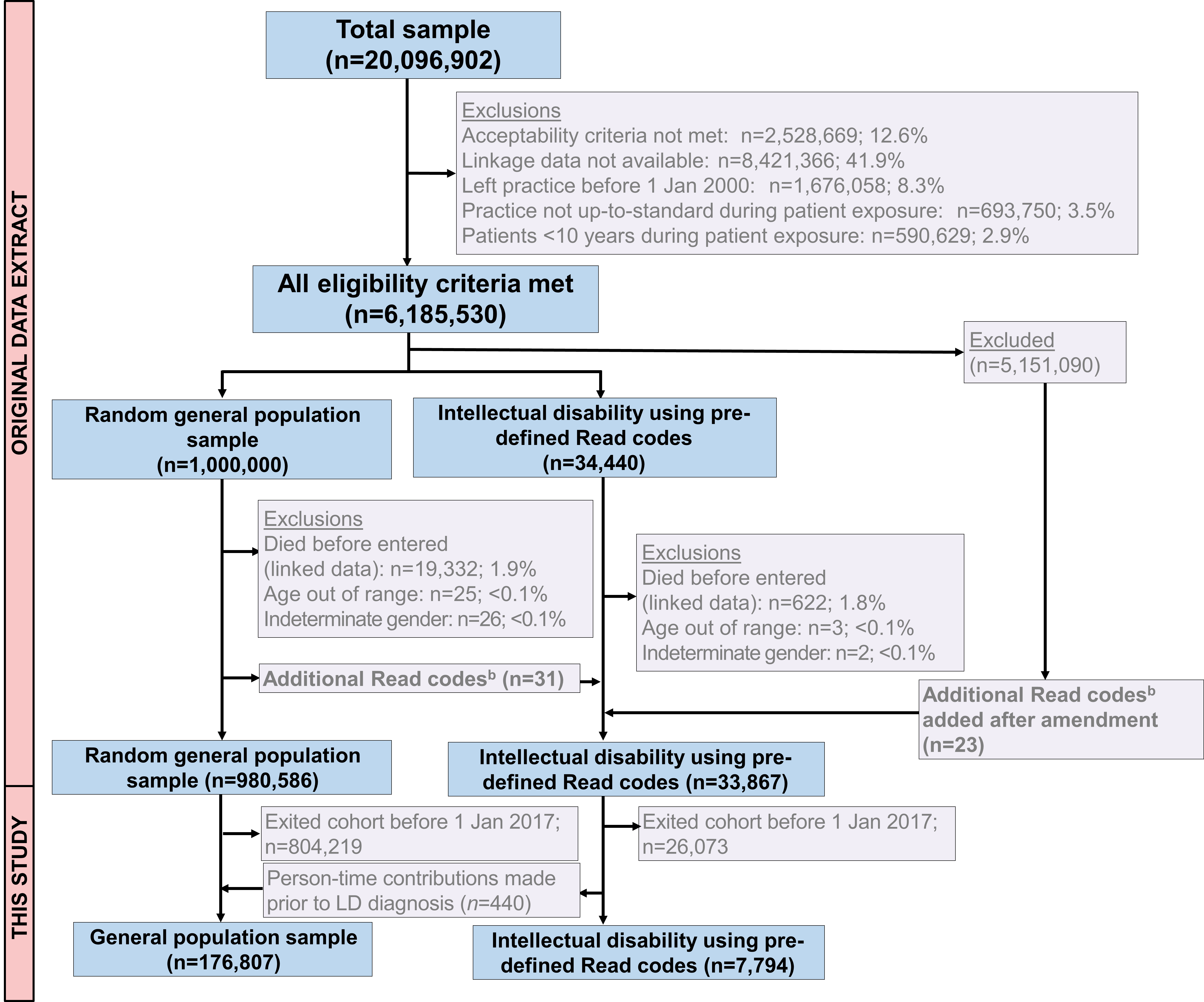
**

^a^ For the original extraction, an unstratified random sample was selected. Age (median 34.73yrs vs 34.74yrs), gender (48.39% vs 48.36%), proportion of deaths (estimated from CPRD data prior to deaths linkage data availability; 7.12% vs 7.13%), and length of time in the cohort (also estimated prior to linkage data availability; 5.10 vs 5.09yrs) for the selected vs entire population were compared to ensure that no obvious differences were evident.

^b^ Additional Read codes for Angelman and Cockayne syndrome added

**Table S2: Diagnostic and classification codes for intellectual disabilities, ethnicity and severe health needs investigated**

| **Primary care Read codes for intellectual disability diagnoses**  (identified from Tests, Referrals or Clinical files) | | |
| --- | --- | --- |
| **Read Code** | **Read Code Description** | |
| C31yX00 | Disorder of glycoprotein metabolism, unspecified | |
| C372000 | Hypoxanthine-guanine-phosphoribosyltransferase deficiency | |
| C372011 | Lesch - Nyhan syndrome | |
| C372300 | Lesch-Nyhan syndrome | |
| C372z00 | Other disorder of purine or pyrimidine metabolism NOS | |
| E141000 | Active disintegrative psychoses | |
| E141100 | Residual disintegrative psychoses | |
| E141z00 | Disintegrative psychosis NOS | |
| Eu70000 | [X]Mld mental retard with statement no or min impairm behav | |
| Eu70100 | [X]Mld mental retard sig impairment behav req attent/treatmt | |
| Eu70y00 | [X]Mild mental retardation, other impairments of behaviour | |
| Eu70z00 | [X]Mild mental retardation without mention impairment behav | |
| Eu71000 | [X]Mod mental retard with statement no or min impairm behav | |
| Eu71100 | [X]Mod mental retard sig impairment behav req attent/treatmt | |
| Eu71y00 | [X]Mod retard oth behav impair | |
| Eu71z00 | [X]Mod mental retardation without mention impairment behav | |
| Eu72000 | [X]Sev mental retard with statement no or min impairm behav | |
| Eu72100 | [X]Sev mental retard sig impairment behav req attent/treatmt | |
| Eu72y00 | [X]Severe mental retardation, other impairments of behaviour | |
| Eu72z00 | [X]Sev mental retardation without mention impairment behav | |
| Eu73000 | [X]Profound ment retrd wth statement no or min impairm behav | |
| Eu73100 | [X]Profound ment retard sig impairmnt behav req attent/treat | |
| Eu73y00 | [X]Profound mental retardation, other impairments of behavr | |
| Eu73z00 | [X]Prfnd mental retardation without mention impairment behav | |
| Eu7y000 | [X]Oth mental retard with statement no or min impairm behav | |
| Eu7y100 | [X]Oth mental retard sig impairment behav req attent/treatmt | |
| Eu7yy00 | [X]Other mental retardation, other impairments of behaviour | |
| Eu7yz00 | [X]Other mental retardation without mention impairment behav | |
| Eu7z000 | [X]Unsp mental retard with statement no or min impairm behav | |
| Eu7z100 | [X]Unsp mentl retard sig impairment behav req attent/treatmt | |
| Eu7zy00 | [X]Unspecified mental retardatn, other impairments of behav | |
| Eu7zz00 | [X]Unsp mental retardation without mention impairment behav | |
| Eu81400 | [X]Moderate learning disability | |
| Eu81500 | [X]Severe learning disability | |
| Eu81600 | [X]Mild learning disability | |
| Eu81700 | [X]Profound learning disability | |
| Eu81800 | [X]Specific learning disability | |
| Eu81z00 | [X]Developmental disorder of scholastic skills, unspecified | |
| Eu81z11 | [X]Learning disability NOS | |
| Eu81z12 | [X]Learning disorder NOS | |
| Eu81z13 | [X]Learn acquisition disab NOS | |
| Eu84112 | [X]Mental retardation with autistic features | |
| Eu84200 | [X]Rett's syndrome | |
| Eu84300 | [X]Other childhood disintegrative disorder | |
| Eu84311 | [X]Dementia infantalis | |
| Eu84312 | [X]Disintegrative psychosis | |
| Eu84313 | [X]Heller's syndrome | |
| Eu84400 | [X]Overactive disorder assoc mental retard/stereotype movts | |
| P22yz00 | Other reduction deformity of brain NOS | |
| PJ33100 | Deletion of long arm of chromosome 18 | |
| PJ33111 | 18p- syndrome | |
| PJ33200 | Deletion of short arm of chromosome 18 | |
| PJ33211 | 18q- syndrome | |
| PJ33300 | Smith-Magenis syndrome | |
| PJ33400 | Jacobsen syndrome | |
| PJ33500 | Greig cephalopolysyndactyly syndrome | |
| PJ33700 | 3p deletion syndrome | |
| PJ33800 | Chromosome 4q deletion syndrome | |
| PJ33900 | Langer-Giedion syndrome | |
| PJ33A00 | Kleefstra syndrome | |
| PJ50000 | Trisomy 6 | |
| PJ50100 | Trisomy 7 | |
| PJ50200 | Trisomy 8 | |
| PJ50300 | Trisomy 9 | |
| PJ50400 | Trisomy 10 | |
| PJ50500 | Trisomy 11 | |
| PJ50600 | Trisomy 12 | |
| PJ50700 | Other trisomy C syndromes | |
| PJ50800 | Trisomy 22 | |
| PJ50w00 | Whole chromosome trisomy, meitotic nondisjunction | |
| PJ50x00 | Whole chromosome trisomy, mosaicism | |
| PJ50x11 | Whole chromosome trisomy, mitotic nondisjunction | |
| PJ50y00 | Other specified whole chromosome trisomy syndrome | |
| PJ50z00 | Whole chromosome trisomy syndrome NOS | |
| PJ51000 | Major partial trisomy | |
| PJ51100 | Minor partial trisomy | |
| PJ51200 | 10q partial trisomy syndrome | |
| PJ51300 | Trisomy 4p syndrome | |
| PJ51400 | Trisomy 9p syndrome | |
| PJ51500 | 15q partial trisomy syndrome | |
| PJ51z00 | Partial trisomy syndrome NOS | |
| PJ52300 | Triploidy | |
| PJ52400 | Polyploidy | |
| PJ52z00 | Trisomy of autosomes NEC NOS | |
| PJyy200 | Fragile X chromosome | |
| PJyy400 | Fragile X syndrome | |
| PKy6100 | Cockayne syndrome | |
| PKy9300 | Prader - Willi syndrome | |
| PKyz.11 | Cockayne's syndrome | |
| PKyz511 | Angelman syndrome | |
| PKyz700 | Angelman's syndrome | |
| Pyu0200 | [X]Other reduction deformities of brain | |
| Pyu0300 | [X]Other specified congenital malformations of brain | |
| PyuA000 | [X]Oth specif trisomies & partial trisomies of autosomes | |
| R034y11 | [D]Global retardation | |
| ZL1B500 | Under care of psychiatrist for mental handicap | |
| 918e.00 | On learning disability register | |
| C031.00 | Goitrous cretin | |
| C0A..00 | Congenital iodine deficiency syndrome | |
| C0A0.00 | Congenital iodine-deficiency syndrome, neurological type | |
| C0A1.00 | Congenital iodine-deficiency syndrome, myxoedematous type | |
| C301.00 | Phenylketonuria | |
| C372.00 | Other disorders of purine and pyrimidine metabolism | |
| C377.00 | Disorders of glycoprotein metabolism | |
| E141.00 | Disintegrative psychosis | |
| E3..00 | Mental retardation | |
| E30..00 | Mild mental retardation, IQ in range 50-70 | |
| E31..00 | Other specified mental retardation | |
| E310.00 | Moderate mental retardation, IQ in range 35-49 | |
| E311.00 | Severe mental retardation, IQ in range 20-34 | |
| E312.00 | Profound mental retardation with IQ less than 20 | |
| E31z.00 | Other specified mental retardation NOS | |
| E3y..00 | Other specified mental retardation | |
| E3z..00 | Mental retardation NOS | |
| Eu7..00 | [X]Mental retardation | |
| Eu70.00 | [X]Mild mental retardation | |
| Eu71.00 | [X]Moderate mental retardation | |
| Eu72.00 | [X]Severe mental retardation | |
| Eu73.00 | [X]Profound mental retardation | |
| Eu7y.00 | [X]Other mental retardation | |
| Eu7z.00 | [X]Unspecified mental retardation | |
| P01..00 | Craniorachischisis | |
| P02..00 | Iniencephaly | |
| P22..00 | Reduction deformities of brain | |
| P224.00 | Arhinencephaly | |
| P225.00 | Holoprosencephaly | |
| P22y.00 | Other specified reduction deformities of brain | |
| P22z.00 | Reduction deformities of brain NOS | |
| PJ0..00 | Down's syndrome - trisomy 21 | |
| PJ00.00 | Trisomy 21, meiotic nondisjunction | |
| PJ02.00 | Trisomy 21, translocation | |
| PJ0z.00 | Down's syndrome NOS | |
| PJ1..00 | Patau's syndrome - trisomy 13 | |
| PJ10.00 | Trisomy 13, meiotic nondisjunction | |
| PJ11.00 | Trisomy 13, mosaicism | |
| PJ12.00 | Trisomy 13, translocation | |
| PJ1z.00 | Patau's syndrome NOS | |
| PJ2..00 | Edward's syndrome - trisomy 18 | |
| PJ20.00 | Trisomy 18, meiotic nondisjunction | |
| PJ21.00 | Trisomy 18, mosaicism | |
| PJ22.00 | Trisomy 18, translocation | |
| PJ2z.00 | Edward's syndrome NOS | |
| PJ30.00 | Antimongolism syndrome | |
| PJ31.00 | Cri-du-chat syndrome | |
| PJ32.00 | Deletion of short arm of chromosome 4 | |
| PJ3z.00 | Monosomies and deletions from the autosomes NOS | |
| PJ50.00 | Whole chromosome trisomy syndromes | |
| PJ51.00 | Partial trisomy syndromes | |
| PJ52.00 | Trisomies of autosomes NEC | |
| PJ9..00 | Mowat-Wilson syndrome | |
| PK5..00 | Tuberous sclerosis | |
| PKy4.00 | William syndrome | |
| ZS34.00 | Developmental disorder of scholastic skill | |
| E3...00 | Mental retardation | |
| C03..11 | Cretinism | |
| C372.11 | Lesch - Nyhan syndrome | |
| E141.11 | Heller's syndrome | |
| E30..11 | Educationally subnormal | |
| E310.11 | Imbecile | |
| E312.11 | Idiocy | |
| Eu70.11 | [X]Feeble-mindedness | |
| Eu71.11 | [X]Moderate mental subnormality | |
| Eu72.11 | [X]Severe mental subnormality | |
| Eu73.11 | [X]Profound mental subnormality | |
| Eu7z.11 | [X]Mental deficiency NOS | |
| PJ0..11 | Mongolism | |
| PJ01.11 | Trisomy 21, mitotic nondisjunction | |
| PJ02.11 | Partial trisomy 21 in Down's syndrome | |
| PJ0z.11 | Trisomy 21 NOS | |
| PJ11.11 | Trisomy 13, mitotic nondisjunction | |
| PJ12.11 | Partial trisomy 13 in Patau's syndrome | |
| PJ1z.11 | Trisomy 13 NOS | |
| PJ21.11 | Trisomy 18, mitotic nondisjunction | |
| PJ22.11 | Partial trisomy 18 in Edward's syndrome | |
| PJ2z.11 | TRISOMY 18 NOS | |
| PJ30.11 | Deletion of long arm of chromosome 21 | |
| PJ31.11 | Deletion of short arm of chromosome 5 | |
| PJ32.11 | Wolff - Hirschorn syndrome | |
| PKy0.11 | Prader-Willi Syndrome | |
| ZS34.11 | Learning disability | |
| C03z.12 | Cretinism | |
| E30..12 | Feeble-minded | |
| Eu70.12 | [X]Mild mental subnormality | |
| Eu7z.12 | [X]Mental subnormality NOS | |
| PJ0..12 | Trisomy 21 | |
| PKy0.12 | Prader-Willi syndrome | |
| E30..13 | Moron | |
| PJ0..13 | Trisomy 22 | |
| 8Ce6.00 | Preferred place of care - learning disability unit | |
| 9HB..00 | Learning disabilities administration status | |
| 9HB0.00 | Learning disabilities health action plan declined | |
| 9HB1.00 | Learning disabilities health action plan offered | |
| 9HB2.00 | Learning disabilities health action plan reviewed | |
| 9HB3.00 | Learning disabilities health assessment | |
| 9HB4.00 | Learning disabilities health action plan completed | |
| 9HB5.00 | Learning disabilities annual health assessment | |
| 9HB6.00 | Learning disabilities annual health assessment declined | |
| 9HB7.00 | Did not attend learning disabilities annual health assessmnt | |
| 9hL..00 | Exception reporting: learning disability quality indicators | |
| 9hL0.00 | Exc learn disability quality indicators: informed dissent | |
| 9hL1.00 | Exc learn disability quality indicators: patient unsuitable | |
| 9mA..00 | Learning disability annual health check invitation | |
| 9mA0.00 | Learning disability annual health check verbal invitation | |
| 9mA1.00 | Learning disability annual health check telephone invitation | |
| 9mA2.00 | Learning disability annual health check letter invitation | |
| 9mA2000 | Learning disability annual health check invtation 1st letter | |
| 9mA2100 | Learning disability annual health check invtation 2nd letter | |
| 9mA2200 | Learning disability annual health check invtation 3rd letter | |
| 9Nh4.00 | Under care of community learning disability team | |
| 9HB6.11 | Learning disabilities annual health check declined | |
| 9HB7.11 | Did not attend learning disabilities annual health check | |
| 13Z3.00 | Low I.Q. | |
| 69DB.00 | Learning disability health examination | |
| 94Z9.00 | Preferred place of death: learning disability unit | |
| 6664 | Mental handicap problem | |
| **Primary care codes for ethnicity status** | | |
| **Read Code** | **Read code description** | **Ethnic group** |
| 916E.00 | Patient ethnicity unknown | Ethnic group not specified |
| 9i...00 | Ethnic category - 2001 census | Ethnic group not specified |
| 9i0..00 | British or mixed British - ethnic category 2001 census | Ethnic group not specified |
| 9i00.00 | White British - ethnic category 2001 census | White |
| 9i1..00 | Irish - ethnic category 2001 census | White |
| 9i10.00 | White Irish - ethnic category 2001 census | White |
| 9i2..00 | Other White background - ethnic category 2001 census | White |
| 9i20.00 | English - ethnic category 2001 census | White |
| 9i21.00 | Scottish - ethnic category 2001 census | White |
| 9i22.00 | Welsh - ethnic category 2001 census | White |
| 9i23.00 | Cornish - ethnic category 2001 census | White |
| 9i24.00 | Northern Irish - ethnic category 2001 census | White |
| 9i25.00 | Ulster Scots - ethnic category 2001 census | White |
| 9i26.00 | Cypriot (part not stated) - ethnic category 2001 census | White |
| 9i27.00 | Greek - ethnic category 2001 census | White |
| 9i28.00 | Greek Cypriot - ethnic category 2001 census | White |
| 9i29.00 | Turkish - ethnic category 2001 census | White |
| 9i2A.00 | Turkish Cypriot - ethnic category 2001 census | White |
| 9i2B.00 | Italian - ethnic category 2001 census | White |
| 9i2C.00 | Irish Traveller - ethnic category 2001 census | White |
| 9i2D.00 | Traveller - ethnic category 2001 census | White |
| 9i2E.00 | Gypsy/Romany - ethnic category 2001 census | White |
| 9i2F.00 | Polish - ethnic category 2001 census | White |
| 9i2G.00 | Baltic Estonian/Latvian/Lithuanian - ethn categ 2001 census | White |
| 9i2H.00 | Commonwealth (Russian) Indep States - ethn categ 2001 census | White |
| 9i2J.00 | Kosovan - ethnic category 2001 census | White |
| 9i2K.00 | Albanian - ethnic category 2001 census | White |
| 9i2L.00 | Bosnian - ethnic category 2001 census | White |
| 9i2M.00 | Croatian - ethnic category 2001 census | White |
| 9i2N.00 | Serbian - ethnic category 2001 census | White |
| 9i2P.00 | Other republics former Yugoslavia - ethnic categ 2001 census | White |
| 9i2Q.00 | Mixed Irish and other White - ethnic category 2001 census | White |
| 9i2R.00 | Oth White European/European unsp/Mixed European 2001 census | White |
| 9i2S.00 | Other mixed White - ethnic category 2001 census | White |
| 9i2T.00 | Other White or White unspecified ethnic category 2001 census | White |
| 9i3..00 | White and Black Caribbean - ethnic category 2001 census | Mixed |
| 9i4..00 | White and Black African - ethnic category 2001 census | Mixed |
| 9i5..00 | White and Asian - ethnic category 2001 census | Mixed |
| 9i6..00 | Other Mixed background - ethnic category 2001 census | Mixed |
| 9i60.00 | Black and Asian - ethnic category 2001 census | Mixed |
| 9i61.00 | Black and Chinese - ethnic category 2001 census | Mixed |
| 9i62.00 | Black and White - ethnic category 2001 census | Mixed |
| 9i63.00 | Chinese and White - ethnic category 2001 census | Mixed |
| 9i64.00 | Asian and Chinese - ethnic category 2001 census | Mixed |
| 9i65.00 | Other Mixed or Mixed unspecified ethnic category 2001 census | Mixed |
| 9i7..00 | Indian or British Indian - ethnic category 2001 census | S.Asian |
| 9i8..00 | Pakistani or British Pakistani - ethnic category 2001 census | S.Asian |
| 9i9..00 | Bangladeshi or British Bangladeshi - ethn categ 2001 census | S.Asian |
| 9iA..00 | Other Asian background - ethnic category 2001 census | S.Asian |
| 9iA1.00 | Punjabi - ethnic category 2001 census | S.Asian |
| 9iA2.00 | Kashmiri - ethnic category 2001 census | S.Asian |
| 9iA3.00 | East African Asian - ethnic category 2001 census | S.Asian |
| 9iA4.00 | Sri Lankan - ethnic category 2001 census | Other ethnic group |
| 9iA5.00 | Tamil - ethnic category 2001 census | Other ethnic group |
| 9iA6.00 | Sinhalese - ethnic category 2001 census | Other ethnic group |
| 9iA7.00 | Caribbean Asian - ethnic category 2001 census | Mixed |
| 9iA8.00 | British Asian - ethnic category 2001 census | S.Asian |
| 9iA9.00 | Mixed Asian - ethnic category 2001 census | S.Asian |
| 9iAA.00 | Other Asian or Asian unspecified ethnic category 2001 census | S.Asian |
| 9iB..00 | Caribbean - ethnic category 2001 census | Black |
| 9iC..00 | African - ethnic category 2001 census | Black |
| 9iD..00 | Other Black background - ethnic category 2001 census | Black |
| 9iD0.00 | Somali - ethnic category 2001 census | Black |
| 9iD1.00 | Nigerian - ethnic category 2001 census | Black |
| 9iD2.00 | Black British - ethnic category 2001 census | Black |
| 9iD3.00 | Mixed Black - ethnic category 2001 census | Black |
| 9iD4.00 | Other Black or Black unspecified ethnic category 2001 census | Black |
| 9iE..00 | Chinese - ethnic category 2001 census | Other ethnic group |
| 9iF..00 | Other - ethnic category 2001 census | Other ethnic group |
| 9iF0.00 | Vietnamese - ethnic category 2001 census | Other ethnic group |
| 9iF1.00 | Japanese - ethnic category 2001 census | Other ethnic group |
| 9iF2.00 | Filipino - ethnic category 2001 census | Other ethnic group |
| 9iF3.00 | Malaysian - ethnic category 2001 census | Other ethnic group |
| 9iF4.00 | Buddhist - ethnic category 2001 census | Other ethnic group |
| 9iF5.00 | Hindu - ethnic category 2001 census | Other ethnic group |
| 9iF6.00 | Jewish - ethnic category 2001 census | Other ethnic group |
| 9iF7.00 | Muslim - ethnic category 2001 census | Other ethnic group |
| 9iF8.00 | Sikh - ethnic category 2001 census | Other ethnic group |
| 9iF9.00 | Arab - ethnic category 2001 census | Other ethnic group |
| 9iFA.00 | North African - ethnic category 2001 census | Other ethnic group |
| 9iFB.00 | Mid East (excl Israeli, Iranian & Arab) - eth cat 2001 cens | Other ethnic group |
| 9iFC.00 | Israeli - ethnic category 2001 census | Other ethnic group |
| 9iFD.00 | Iranian - ethnic category 2001 census | Other ethnic group |
| 9iFE.00 | Kurdish - ethnic category 2001 census | Other ethnic group |
| 9iFF.00 | Moroccan - ethnic category 2001 census | Other ethnic group |
| 9iFG.00 | Latin American - ethnic category 2001 census | Other ethnic group |
| 9iFH.00 | South and Central American - ethnic category 2001 census | Other ethnic group |
| 9iFJ.00 | Mauritian/Seychellois/Maldivian/St Helena eth cat 2001census | Other ethnic group |
| 9iFK.00 | Any other group - ethnic category 2001 census | Other ethnic group |
| 9iG..00 | Ethnic category not stated - 2001 census | Ethnic group not specified |
| 9S...00 | Ethnic groups (1991 census) | Ethnic group not specified |
| 9S1..00 | White | White |
| 9S10.00 | White British | White |
| 9S11.00 | White Irish | White |
| 9S12.00 | Other white ethnic group | White |
| 9S13.00 | White Scottish | White |
| 9S14.00 | Other white British ethnic group | White |
| 9S2..00 | Black Caribbean | Black |
| 9S3..00 | Black African | Black |
| 9S4..00 | Black, other, non-mixed origin | Black |
| 9S41.00 | Black British | Black |
| 9S42.00 | Black Caribbean/W.I./Guyana | Black |
| 9S42.11 | Black Caribbean | Black |
| 9S42.12 | Black West Indian | Black |
| 9S42.13 | Black Guyana | Black |
| 9S43.00 | Black N African/Arab/Iranian | Black |
| 9S43.11 | Black North African | Black |
| 9S43.12 | Black Arab | Black |
| 9S43.13 | Black Iranian | Black |
| 9S44.00 | Black - other African country | Black |
| 9S45.00 | Black E Afric Asia/Indo-Caribb | Mixed |
| 9S45.11 | Black East African Asian | Mixed |
| 9S45.12 | Black Indo-Caribbean | Mixed |
| 9S46.00 | Black Indian sub-continent | Mixed |
| 9S47.00 | Black - other Asian | Mixed |
| 9S48.00 | Black Black - other | Black |
| 9S5..00 | Black - other, mixed | Black |
| 9S51.00 | Other Black - Black/White orig | Mixed |
| 9S52.00 | Other Black - Black/Asian orig | Mixed |
| 9S6..00 | Indian | S.Asian |
| 9S7..00 | Pakistani | S.Asian |
| 9S8..00 | Bangladeshi | S.Asian |
| 9S9..00 | Chinese | Other ethnic group |
| 9SA..00 | Other ethnic non-mixed (NMO) | Other ethnic group |
| 9SA1.00 | Brit. ethnic minor. spec.(NMO) | Other ethnic group |
| 9SA2.00 | Brit. ethnic minor. unsp (NMO) | Other ethnic group |
| 9SA3.00 | Caribbean I./W.I./Guyana (NMO) | Black |
| 9SA3.11 | Caribbean Island (NMO) | Black |
| 9SA3.12 | West Indian (NMO) | Black |
| 9SA3.13 | Guyana (NMO) | Black |
| 9SA4.00 | N African Arab/Iranian (NMO) | Other ethnic group |
| 9SA4.11 | North African Arab (NMO) | Other ethnic group |
| 9SA4.12 | Iranian (NMO) | Other ethnic group |
| 9SA5.00 | Other African countries (NMO) | Black |
| 9SA6.00 | E Afric Asian/Indo-Carib (NMO) | Mixed |
| 9SA6.11 | East African Asian (NMO) | S.Asian |
| 9SA7.00 | Indian sub-continent (NMO) | S.Asian |
| 9SA8.00 | Other Asian (NMO) | S.Asian |
| 9SA9.00 | Irish (NMO) | White |
| 9SAA.00 | Greek/Greek Cypriot (NMO) | White |
| 9SAA.11 | Greek (NMO) | White |
| 9SAA.12 | Greek Cypriot (NMO) | White |
| 9SAB.00 | Turkish/Turkish Cypriot (NMO) | White |
| 9SAB.11 | Turkish (NMO) | White |
| 9SAB.12 | Turkish Cypriot (NMO) | White |
| 9SAC.00 | Other European (NMO) | White |
| 9SAD.00 | Other ethnic NEC (NMO) | Other ethnic group |
| 9SB..00 | Other ethnic, mixed origin | Mixed |
| 9SB1.00 | Other ethnic, Black/White orig | Mixed |
| 9SB2.00 | Other ethnic, Asian/White orig | Mixed |
| 9SB3.00 | Other ethnic, mixed white orig | White |
| 9SB4.00 | Other ethnic, other mixed orig | Mixed |
| 9SB5.00 | Black Caribbean and White | Mixed |
| 9SB6.00 | Black African and White | Mixed |
| 9SC..00 | Vietnamese | Other ethnic group |
| 9SD..00 | Ethnic group not given - patient refused | Ethnic group not specified |
| 9SE..00 | Ethnic group not recorded | Ethnic group not specified |
| 9SG..00 | Other black ethnic group | Black |
| 9SH..00 | Other Asian ethnic group | S.Asian |
| 9SI..00 | Irish traveller | White |
| 9SJ..00 | Other ethnic group | Other ethnic group |
| 9SZ..00 | Ethnic groups (census) NOS | Ethnic group not specified |
| 9T...00 | Ethnicity and other related nationality data | Ethnic group not specified |
| 9t0..00 | Ethnic category - 2011 census England and Wales | Ethnic group not specified |
| 9t00.00 | White:Eng/Welsh/Scot/NI/Brit - England and Wales 2011 census | White |
| 9t01.00 | White: Irish - England and Wales ethnic category 2011 census | White |
| 9t02.00 | White: Gypsy/Irish Traveller - Eng+Wales eth cat 2011 census | White |
| 9t03.00 | White: other White backgrd- Eng+Wales ethnic cat 2011 census | White |
| 9t04.00 | Mixed: White+Black Caribbean - Eng+Wales eth cat 2011 census | Mixed |
| 9t05.00 | Mixed: White+Black African - Eng+Wales eth cat 2011 census | Mixed |
| 9t06.00 | Mixed: White+Asian - Eng+Wales ethnic category 2011 census | Mixed |
| 9t08.00 | Asian/Asian Brit: Indian - Eng+Wales ethnic cat 2011 census | S.Asian |
| 9t09.00 | Asian/Asian British:Pakistani- Eng+Wales eth cat 2011 census | S.Asian |
| 9t0A.00 | Asian/Asian Brit: Bangladeshi- Eng+Wales eth cat 2011 census | S.Asian |
| 9t0B.00 | Asian/Asian Brit: Chinese - Eng+Wales ethnic cat 2011 census | Other ethnic group |
| 9t0C.00 | Asian/Asian Brit: other Asian- Eng+Wales eth cat 2011 census | S.Asian |
| 9t0D.00 | Black/African/Carib/Black Brit: African- Eng+Wales 2011 cens | Black |
| 9t0E.00 | Black/African/Caribbn/Black Brit: Caribbean - Eng+Wales 2011 | Black |
| 9t0F.00 | Black/Afr/Carib/Black Brit: other Black- Eng+Wales 2011 cens | Black |
| 9t0G.00 | Other ethnic group: Arab - Eng+Wales ethnic cat 2011 census | Other ethnic group |
| 9t0H.00 | Other ethnic: any other grp - Eng+Wales eth cat 2011 census | Other ethnic group |
| 9T1..00 | New Zealand ethnic groups | White |
| 9t12.00 | Mixed: White and Black Caribbean - NI ethnic cat 2011 census | Mixed |
| 9t13.00 | Mixed: White and Black African - NI ethnic cat 2011 census | Mixed |
| 9t14.00 | Mixed: White and Asian - NI ethnic category 2011 census | Mixed |
| 9t15.00 | Mixed: other Mixed/multiple ethnic backgrd - NI 2011 census | Mixed |
| 9t16.00 | Asian or Asian British: Indian - NI ethnic cat 2011 census | S.Asian |
| 9t17.00 | Asian/Asian British: Pakistani - NI ethnic cat 2011 census | S.Asian |
| 9t18.00 | Asian/Asian British: Bangladeshi - NI ethnic cat 2011 census | S.Asian |
| 9t19.00 | Asian/Asian British: Chinese - NI ethnic cat 2011 census | Other ethnic group |
| 9T1A.00 | Other Pacific ethnic group | Other ethnic group |
| 9t1E.00 | Other ethnic group: Arab - NI ethnic category 2011 census | Other ethnic group |
| 9t1F.00 | Other ethnic group: any other grp- NI ethnic cat 2011 census | Other ethnic group |
| 9T1Y.00 | Other New Zealand ethnic group | White |
| 9T1Z.00 | New Zealand ethnic group NOS | White |
| 9t2..00 | Ethnic category - 2011 census Scotland | Ethnic group not specified |
| 9t20.00 | White: Scottish - Scotland ethnic category 2011 census | White |
| 9t21.00 | White: other British - Scotland ethnic category 2011 census | White |
| 9t22.00 | White: Irish - Scotland ethnic category 2011 census | White |
| 9t24.00 | White: Polish - Scotland ethnic category 2011 census | White |
| 9t25.00 | White: other White ethnic grp- Scotland ethnic cat 2011 cens | White |
| 9t26.00 | Mixed/multiple ethnic grps: any- Scot ethnic cat 2011 census | Mixed |
| 9t27.00 | Asian: Pakistani/Pakistani Scot/Pakistani Brit- Scot 2011 | S.Asian |
| 9t28.00 | Asian: Indian, Indian Scot/Indian Brit- Scotland 2011 census | S.Asian |
| 9t29.00 | Bangladeshi, Bangladeshi Scot or Bangladeshi Brit- Scot 2011 | S.Asian |
| 9t2A.00 | Asian: Chinese - Scotland ethnic category 2011 census | Other ethnic group |
| 9t2B.00 | Asian: other Asian group - Scotland ethnic cat 2011 census | S.Asian |
| 9t2C.00 | African: African/African Scot/African Brit - Scotland 2011 | Black |
| 9t2D.00 | African: any other African - Scotland ethnic cat 2011 census | Black |
| 9t2G.00 | Carib/Black: any other Black/Caribbean grp - Scotland 2011 | Black |
| 9t2H.00 | Other ethnic grp: Arab/Arab Scot/Arab British- Scotland 2011 | Other ethnic group |
| 9t2J.00 | Other ethnic grp: any other ethnic grp- Scotland 2011 census | Other ethnic group |
| **Free-text field from Hospital Episode Statistics’ patient file** | | |
| **Free text** | **Ethnic group** | |
| Bangladeshi | S.Asian | |
| Bl_Afric | Black | |
| Bl_Carib | Black | |
| Bl_Other | Black | |
| Chinese | Other ethnic group | |
| Indian | S.Asian | |
| Mixed | Mixed | |
| Oth_Asian | S.Asian | |
| Other | Other ethnic group | |
| Pakistani | S.Asian | |
| Unknown | Ethnic group not specified | |
| White | White | |
| **Primary care Read codes for severe health needs**  (identified from Tests, Referrals or Clinical files) | | |
| **Read Code** | **Read Code Description** | **Interpretation** |
| 2822.00 | O/E - grand mal fit | Epilepsy |
| 2826.00 | O/E - salaam attack | Epilepsy |
| 6671.00 | Initial epilepsy assessment | Epilepsy |
| 6672.00 | Follow-up epilepsy assessment | Epilepsy |
| 6674.00 | Epilepsy associated problems | Epilepsy |
| 6677.00 | Epilepsy drug side effects | Epilepsy |
| 6678.00 | Epilepsy treatment changed | Epilepsy |
| 6679.00 | Epilepsy treatment started | Epilepsy |
| 14On.00 | At risk of sudden unexpected death in epilepsy | Epilepsy |
| 1B1W.00 | Transient epileptic amnesia | Epilepsy |
| 1B26.00 | Trigger factor for seizure | Epilepsy |
| 1O30.00 | Epilepsy confirmed | Epilepsy |
| 44W4.11 | Epilim: blood level | Epilepsy |
| 667..00 | Epilepsy monitoring | Epilepsy |
| 667B.00 | Nocturnal epilepsy | Epilepsy |
| 667D.00 | Epilepsy control poor | Epilepsy |
| 667E.00 | Epilepsy care arrangement | Epilepsy |
| 667G.00 | Epilepsy restricts employment | Epilepsy |
| 667H.00 | Epilepsy prevents employment | Epilepsy |
| 667J.00 | Epilepsy impairs education | Epilepsy |
| 667K.00 | Epilepsy limits activities | Epilepsy |
| 667M.00 | Epilepsy management plan given | Epilepsy |
| 667N.00 | Epilepsy severity | Epilepsy |
| 667R.00 | 2 to 4 seizures a month | Epilepsy |
| 667S.00 | 1 to 7 seizures a week | Epilepsy |
| 667T.00 | Daily seizures | Epilepsy |
| 667V.00 | Many seizures a day | Epilepsy |
| 667W.00 | Emergency epilepsy treatment since last appointment | Epilepsy |
| 667Z.00 | Epilepsy monitoring NOS | Epilepsy |
| 9Of3.00 | Epilepsy monitoring verbal invite | Epilepsy |
| 9Of4.00 | Epilepsy monitoring telephone invite | Epilepsy |
| 9Of5.00 | Epilepsy monitoring call first letter | Epilepsy |
| 9Of6.00 | Epilepsy monitoring call second letter | Epilepsy |
| 9Of7.00 | Epilepsy monitoring call third letter | Epilepsy |
| Eu05212 | [X]Schizophrenia-like psychosis in epilepsy | Epilepsy |
| Eu05y11 | [X]Epileptic psychosis NOS | Epilepsy |
| Eu06013 | [X]Limbic epilepsy personality | Epilepsy |
| Eu80300 | [X]Acquired aphasia with epilepsy [Landau - Kleffner] | Epilepsy |
| F132100 | Progressive myoclonic epilepsy | Epilepsy |
| F132z12 | Myoclonic seizure | Epilepsy |
| F25..00 | Epilepsy | Epilepsy |
| F250.00 | Generalised nonconvulsive epilepsy | Epilepsy |
| F250200 | Epileptic seizures - atonic | Epilepsy |
| F250300 | Epileptic seizures - akinetic | Epilepsy |
| F250500 | Lennox-Gastaut syndrome | Epilepsy |
| F250y00 | Other specified generalised nonconvulsive epilepsy | Epilepsy |
| F250z00 | Generalised nonconvulsive epilepsy NOS | Epilepsy |
| F251.00 | Generalised convulsive epilepsy | Epilepsy |
| F251000 | Grand mal (major) epilepsy | Epilepsy |
| F251011 | Tonic-clonic epilepsy | Epilepsy |
| F251100 | Neonatal myoclonic epilepsy | Epilepsy |
| F251111 | Otohara syndrome | Epilepsy |
| F251200 | Epileptic seizures - clonic | Epilepsy |
| F251300 | Epileptic seizures - myoclonic | Epilepsy |
| F251400 | Epileptic seizures - tonic | Epilepsy |
| F251500 | Tonic-clonic epilepsy | Epilepsy |
| F251600 | Grand mal seizure | Epilepsy |
| F251y00 | Other specified generalised convulsive epilepsy | Epilepsy |
| F251z00 | Generalised convulsive epilepsy NOS | Epilepsy |
| F252.00 | Petit mal status | Epilepsy |
| F253.00 | Grand mal status | Epilepsy |
| F253.11 | Status epilepticus | Epilepsy |
| F254.00 | Partial epilepsy with impairment of consciousness | Epilepsy |
| F254000 | Temporal lobe epilepsy | Epilepsy |
| F254100 | Psychomotor epilepsy | Epilepsy |
| F254200 | Psychosensory epilepsy | Epilepsy |
| F254300 | Limbic system epilepsy | Epilepsy |
| F254400 | Epileptic automatism | Epilepsy |
| F254500 | Complex partial epileptic seizure | Epilepsy |
| F254z00 | Partial epilepsy with impairment of consciousness NOS | Epilepsy |
| F255.00 | Partial epilepsy without impairment of consciousness | Epilepsy |
| F255000 | Jacksonian, focal or motor epilepsy | Epilepsy |
| F255011 | Focal epilepsy | Epilepsy |
| F255012 | Motor epilepsy | Epilepsy |
| F255100 | Sensory induced epilepsy | Epilepsy |
| F255200 | Somatosensory epilepsy | Epilepsy |
| F255300 | Visceral reflex epilepsy | Epilepsy |
| F255311 | Partial epilepsy with autonomic symptoms | Epilepsy |
| F255400 | Visual reflex epilepsy | Epilepsy |
| F255500 | Unilateral epilepsy | Epilepsy |
| F255600 | Simple partial epileptic seizure | Epilepsy |
| F255y00 | Partial epilepsy without impairment of consciousness OS | Epilepsy |
| F255z00 | Partial epilepsy without impairment of consciousness NOS | Epilepsy |
| F256.11 | Lightning spasms | Epilepsy |
| F256.12 | West syndrome | Epilepsy |
| F256000 | Hypsarrhythmia | Epilepsy |
| F256100 | Salaam attacks | Epilepsy |
| F257.00 | Kojevnikov's epilepsy | Epilepsy |
| F258.00 | Post-ictal state | Epilepsy |
| F259.00 | Early infant epileptic encephalopathy wth suppression bursts | Epilepsy |
| F259.11 | Ohtahara syndrome | Epilepsy |
| F25A.00 | Juvenile myoclonic epilepsy | Epilepsy |
| F25F.00 | Photosensitive epilepsy | Epilepsy |
| F25G.00 | Severe myoclonic epilepsy in infancy | Epilepsy |
| F25H.00 | Generalised seizure | Epilepsy |
| F25X.00 | Status epilepticus, unspecified | Epilepsy |
| F25y.00 | Other forms of epilepsy | Epilepsy |
| F25y000 | Cursive (running) epilepsy | Epilepsy |
| F25y100 | Gelastic epilepsy | Epilepsy |
| F25y200 | Locl-rlt(foc)(part)idiop epilep&epilptic syn seiz locl onset | Epilepsy |
| F25y300 | Complex partial status epilepticus | Epilepsy |
| F25y500 | Panayiotopoulos syndrome | Epilepsy |
| F25yz00 | Other forms of epilepsy NOS | Epilepsy |
| F25z.00 | Epilepsy NOS | Epilepsy |
| F25z.11 | Fit (in known epileptic) NOS | Epilepsy |
| Fyu5000 | [X]Other generalized epilepsy and epileptic syndromes | Epilepsy |
| Fyu5100 | [X]Other epilepsy | Epilepsy |
| Fyu5200 | [X]Other status epilepticus | Epilepsy |
| Fyu5900 | [X]Status epilepticus, unspecified | Epilepsy |
| PK5..12 | Epiloia | Epilepsy |
| R003400 | [D]Nocturnal seizure | Epilepsy |
| SC20000 | Traumatic epilepsy | Epilepsy |
| ZS82.00 | Acquired epileptic aphasia | Epilepsy |
| 3930.00 | Bowels: incontinent | Incontinence |
| 3931.00 | Bowels: occasional accident | Incontinence |
| 3940.00 | Bladder: incontinent | Incontinence |
| 3941.00 | Bladder: occasional accident | Incontinence |
| 16F..00 | Double incontinence | Incontinence |
| 19E2.00 | Soiling - encopresis | Incontinence |
| 19E2.11 | Encopresis symptom | Incontinence |
| 19E2.12 | Soiling symptom | Incontinence |
| 19E3.00 | Incontinent of faeces | Incontinence |
| 19E3.11 | Incontinent of faeces symptom | Incontinence |
| 1A22.00 | Enuresis | Incontinence |
| 1A22000 | Nocturnal enuresis | Incontinence |
| 1A22011 | Bedwetting | Incontinence |
| 1A22100 | Daytime enuresis | Incontinence |
| 1A23.00 | Incontinence of urine | Incontinence |
| 1A24.00 | Stress incontinence | Incontinence |
| 1A24.11 | Stress incontinence - symptom | Incontinence |
| 1A26.00 | Urge incontinence of urine | Incontinence |
| 7B33800 | Insertion retropubic device stress urinary incontinence NEC | Incontinence |
| 7B33C00 | Insertion retropubic dev fem stress urinary incontinence NEC | Incontinence |
| 7B42111 | Insertion of Kaufman prosthesis for male incontinence | Incontinence |
| 7B42113 | Insertion of Rosen prosthesis for male incontinence | Incontinence |
| 8C14.00 | Incontinence care | Incontinence |
| 8D7..12 | Incontinence control | Incontinence |
| 8D71.00 | Incontinence control | Incontinence |
| 8D73.00 | Nocturnal bladder warning syst | Incontinence |
| 8D73.11 | Enuretic alarm | Incontinence |
| 8D73.12 | Enuresis alarm | Incontinence |
| 8HTt.00 | Referral to enuresis clinic | Incontinence |
| 8HTX.00 | Referral to incontinence clinic | Incontinence |
| 8O6..00 | Enuresis support | Incontinence |
| 9No6.00 | Seen in enuresis clinic | Incontinence |
| E276.00 | Non-organic enuresis | Incontinence |
| E276000 | Non-organic primary enuresis | Incontinence |
| E276100 | Non-organic secondary enuresis | Incontinence |
| E276z00 | Non-organic enuresis NOS | Incontinence |
| E277.00 | Non-organic encopresis | Incontinence |
| E277000 | Non-organic continuous encopresis | Incontinence |
| E277z00 | Non-organic encopresis NOS | Incontinence |
| Eu9y000 | [X]Nonorganic enuresis | Incontinence |
| Eu9y100 | [X]Nonorganic encopresis | Incontinence |
| F246112 | Neurogenic bladder | Incontinence |
| K16V011 | Neurogenic bladder | Incontinence |
| K198.00 | Stress incontinence | Incontinence |
| K586.00 | Stress incontinence - female | Incontinence |
| Kyu5A00 | [X]Other specified urinary incontinence | Incontinence |
| M129500 | Incontinence-associated dermatitis | Incontinence |
| R076.00 | [D]Incontinence of faeces | Incontinence |
| R076000 | [D]Encopresis NOS | Incontinence |
| R076100 | [D]Sphincter ani incontinence | Incontinence |
| R076z00 | [D]Incontinence of faeces NOS | Incontinence |
| R083.00 | [D]Incontinence of urine | Incontinence |
| R083000 | [D]Enuresis NOS | Incontinence |
| R083100 | [D]Urethral sphincter incontinence | Incontinence |
| R083200 | [D] Urge incontinence | Incontinence |
| R083z00 | [D]Incontinence of urine NOS | Incontinence |
| Z1J..00 | Procedures to aid continence | Incontinence |
| Z9EA.00 | Provision of incontinence appliance | Incontinence |
| Z9EA100 | Provision of nocturnal bladder warning system | Incontinence |
| Z9EA111 | Provision of enuresis alarm | Incontinence |
| Z9EA112 | Provision of enuretic alarm | Incontinence |
| Z9MO.00 | Enuresis support | Incontinence |
| ZQ3C.00 | Bowels incontinence assessment | Incontinence |
| 6688.00 | Registered partially sighted | Severe visual impairment |
| 6688.11 | Registered partially blind | Severe visual impairment |
| 6689.00 | Registered blind | Severe visual impairment |
| 6689.11 | Registered severely sight impaired | Severe visual impairment |
| 6689.11 | Registered severely sight impaired | Severe visual impairment |
| 7202.00 | Insertion of prosthesis of eye | Severe visual impairment |
| 7203.00 | Attention to prosthesis of eye | Severe visual impairment |
| 7202100 | Insertion of prosthetic replacement for eyeball | Severe visual impairment |
| 7203000 | Revision of prosthetic replacement for orbit | Severe visual impairment |
| 7203100 | Revision of prosthetic replacement for eyeball | Severe visual impairment |
| 7203300 | Removal of prosthetic replacement for eyeball | Severe visual impairment |
| 14T1.00 | H/O: artificial eyeglobe | Severe visual impairment |
| 1a00000 | Uses guide dog for the blind | Severe visual impairment |
| 1B75.00 | Loss of vision | Severe visual impairment |
| 22E6.00 | O/E - glass (prosthetic) eye | Severe visual impairment |
| 22E6.11 | O/E - false eye | Severe visual impairment |
| 22E6.12 | O/E - glass eye | Severe visual impairment |
| 22E6.13 | O/E - prosthetic eye | Severe visual impairment |
| 22EF.00 | O/E - has one eye | Severe visual impairment |
| 2B69.00 | O/E -R-eye counts fingers only | Severe visual impairment |
| 2B6A.00 | O/E-R-eye perceives light only | Severe visual impairment |
| 2B6A.11 | O/E - blind R-eye | Severe visual impairment |
| 2B6B.00 | O/E - R-eye completely blind | Severe visual impairment |
| 2B6C.00 | O/E - R-eye sees hand movements | Severe visual impairment |
| 2B6P.00 | O/E - pinhole R-eye sees hand movements | Severe visual impairment |
| 2B6Q.00 | O/E - pinhole R-eye counts fingers only | Severe visual impairment |
| 2B6R.00 | O/E - pinhole R-eye perceives light only | Severe visual impairment |
| 2B6S.00 | O/E - pinhole R-eye completely blind | Severe visual impairment |
| 2B6T.00 | O/E - R-eye visual acuity (corrected) 1/60 | Severe visual impairment |
| 2B6V.00 | O/E - R-eye visual acuity (corrected) 2/60 | Severe visual impairment |
| 2B6W.00 | O/E - R-eye visual acuity (corrected) 4/60 | Severe visual impairment |
| 2B6X.00 | O/E - R-eye visual acuity (corrected) 5/60 | Severe visual impairment |
| 2B79.00 | O/E -L-eye counts fingers only | Severe visual impairment |
| 2B7A.00 | O/E-L-eye perceives light only | Severe visual impairment |
| 2B7A.11 | O/E - blind L-eye | Severe visual impairment |
| 2B7B.00 | O/E - L-eye completely blind | Severe visual impairment |
| 2B7C.00 | O/E - L-eye sees hand movements | Severe visual impairment |
| 2B7P.00 | O/E - pinhole L-eye sees hand movements | Severe visual impairment |
| 2B7Q.00 | O/E - pinhole L-eye counts fingers only | Severe visual impairment |
| 2B7R.00 | O/E - pinhole L-eye perceives light only | Severe visual impairment |
| 2B7S.00 | O/E - pinhole L-eye completely blind | Severe visual impairment |
| 2B7T.00 | O/E - L-eye visual acuity (corrected) 1/60 | Severe visual impairment |
| 2B7V.00 | O/E - L-eye visual acuity (corrected) 2/60 | Severe visual impairment |
| 2B7W.00 | O/E - L-eye visual acuity (corrected) 4/60 | Severe visual impairment |
| 2B7X.00 | O/E - L-eye visual acuity (corrected) 5/60 | Severe visual impairment |
| 2BBr.00 | Impaired vision due to diabetic retinopathy | Severe visual impairment |
| 668B.00 | Poor visual acuity | Severe visual impairment |
| 668C.00 | Certificate of vision impairment | Severe visual impairment |
| 668D.00 | Registered sight impaired | Severe visual impairment |
| 7202y00 | Other specified insertion of prosthesis of eye | Severe visual impairment |
| 7202z00 | Insertion of prosthesis of eye NOS | Severe visual impairment |
| 7203y00 | Other specified attention to prosthesis of eye | Severe visual impairment |
| 7203z00 | Attention to prosthesis of eye NOS | Severe visual impairment |
| 8D3..00 | Visual aid | Severe visual impairment |
| 8D3..13 | Visual aid provision | Severe visual impairment |
| 8D31.00 | Physiolog. visual assistance | Severe visual impairment |
| 8D36.00 | Removable artificial eye | Severe visual impairment |
| 8D3Z.00 | Visual aid NOS | Severe visual impairment |
| 8E1..00 | Visual defect-remedial therapy | Severe visual impairment |
| 8E1Z.00 | Visual defect-remed.therap.NOS | Severe visual impairment |
| 8F6..11 | Blind rehabilitation | Severe visual impairment |
| 8F61.00 | Blind rehabilitation | Severe visual impairment |
| 8F62.00 | Blind lead dog rehabilitation | Severe visual impairment |
| 8HlE.00 | Referral to visual impairment multidisciplinary team | Severe visual impairment |
| 8O0D.00 | Provision of support as Braille user | Severe visual impairment |
| 9m08.00 | Excluded from diabetic retinopathy screening as blind | Severe visual impairment |
| 9Nfa.00 | Requires deafblind manual alphabet interpreter | Severe visual impairment |
| 9NfB.00 | Requires deafblind communicator guide | Severe visual impairment |
| 9Nfb.00 | Requires deafblind block alphabet interpreter | Severe visual impairment |
| 9NfM.00 | Requires information in contracted (Grade 2) Braille | Severe visual impairment |
| 9NfN.00 | Requires information in uncontracted (Grade 1) Braille | Severe visual impairment |
| 9NlD.00 | Seen by visual impairment teacher | Severe visual impairment |
| 9NnR.00 | Visual frame sign language interpreter needed | Severe visual impairment |
| F101200 | Spielmeyer-Vogt (Batten) disease | Severe visual impairment |
| F404100 | Blind hypotensive eye | Severe visual impairment |
| F404200 | Blind hypertensive eye | Severe visual impairment |
| F49..00 | Blindness and low vision | Severe visual impairment |
| F49..11 | Impaired vision | Severe visual impairment |
| F49..12 | Low vision | Severe visual impairment |
| F49..13 | Partial sight | Severe visual impairment |
| F49..14 | Sight impaired | Severe visual impairment |
| F490.00 | Blindness, both eyes | Severe visual impairment |
| F490000 | Unspecified blindness both eyes | Severe visual impairment |
| F490100 | Both eyes total visual impairment | Severe visual impairment |
| F490200 | Better eye: near total VI, Lesser eye: unspecified | Severe visual impairment |
| F490400 | Better eye: near total VI, Lesser eye: near total VI | Severe visual impairment |
| F490600 | Better eye: profound VI, Lesser eye: total VI | Severe visual impairment |
| F490900 | Acquired blindness, both eyes | Severe visual impairment |
| F490z00 | Blindness both eyes NOS | Severe visual impairment |
| F491.00 | Better eye: low vision, Lesser eye: profound VI | Severe visual impairment |
| F491000 | One eye blind, one eye low vision | Severe visual impairment |
| F491100 | Better eye: severe VI, Lesser eye: blind, unspecified | Severe visual impairment |
| F491300 | Better eye: severe VI, Lesser eye: near total VI | Severe visual impairment |
| F491400 | Better eye: severe VI, Lesser eye: profound VI | Severe visual impairment |
| F491500 | Better eye: moderate VI, Lesser eye: blind, unspecified | Severe visual impairment |
| F491700 | Better eye: moderate VI, Lesser eye: near total VI | Severe visual impairment |
| F491z00 | One eye blind, one eye low vision NOS | Severe visual impairment |
| F492.00 | Low vision, both eyes | Severe visual impairment |
| F492000 | Low vision, both eyes unspecified | Severe visual impairment |
| F492200 | Better eye: severe VI, Lesser eye: severe VI | Severe visual impairment |
| F492300 | Better eye: moderate VI, Lesser eye: low vision unspecified | Severe visual impairment |
| F492400 | Better eye: moderate VI, Lesser eye: severe VI | Severe visual impairment |
| F492500 | Better eye: moderate VI, Lesser eye: moderate VI | Severe visual impairment |
| F492z00 | Low vision, both eyes NOS | Severe visual impairment |
| F493.00 | Visual loss, both eyes unqualified | Severe visual impairment |
| F494.00 | Legal blindness USA | Severe visual impairment |
| F495.00 | Profound impairment, one eye | Severe visual impairment |
| F495000 | Blindness, one eye, unspecified | Severe visual impairment |
| F495100 | Lesser eye: total visual impairment, Better eye: unspecified | Severe visual impairment |
| F495200 | Lesser eye: total VI, Better eye: near normal vision | Severe visual impairment |
| F495300 | Lesser eye: total VI, Better eye: normal vision | Severe visual impairment |
| F495400 | Lesser eye: near total VI, Better eye: unspecified | Severe visual impairment |
| F495500 | Lesser eye: near total VI, Better eye: near normal vision | Severe visual impairment |
| F495600 | Lesser eye: near total VI, Better eye: normal vision | Severe visual impairment |
| F495800 | Lesser eye: profound VI, Better eye: near normal vision | Severe visual impairment |
| F495A00 | Acquired blindness, one eye | Severe visual impairment |
| F495z00 | Profound impairment one eye NOS | Severe visual impairment |
| F496.00 | Low vision, one eye | Severe visual impairment |
| F496000 | Low vision, one eye, unspecified | Severe visual impairment |
| F496100 | Lesser eye: severe VI, Better eye: unspecified | Severe visual impairment |
| F496200 | Lesser eye: severe VI, Better eye: near normal vision | Severe visual impairment |
| F496300 | Lesser eye: severe VI, Better eye: normal vision | Severe visual impairment |
| F496400 | Lesser eye: moderate VI, Better eye: unspecified | Severe visual impairment |
| F496500 | Lesser eye: moderate VI, Better eye: near normal vision | Severe visual impairment |
| F496600 | Lesser eye: moderate VI, Better eye: normal vision | Severe visual impairment |
| F496z00 | Low vision, one eye NOS | Severe visual impairment |
| F497.00 | Severe visual impairment, binocular | Severe visual impairment |
| F498.00 | Moderate visual impairment, binocular | Severe visual impairment |
| F49A.00 | Blindness, monocular | Severe visual impairment |
| F49B.00 | Severe visual impairment, monocular | Severe visual impairment |
| F49C.00 | Moderate visual impairment, monocular | Severe visual impairment |
| F49D.00 | Visual impairment | Severe visual impairment |
| F49y.00 | Visual loss, one eye, unqualified | Severe visual impairment |
| F49z.00 | Visual loss NOS | Severe visual impairment |
| F49z.11 | Acquired blindness | Severe visual impairment |
| F49z000 | Charles Bonnet syndrome | Severe visual impairment |
| F4H..11 | Visual path disorder | Severe visual impairment |
| F4H6.00 | Other visual pathway disorder | Severe visual impairment |
| F4H6100 | Visual pathway disorder due to vascular disorder | Severe visual impairment |
| F4H6z00 | Other visual pathway disorder NOS | Severe visual impairment |
| F4H7.00 | Visual cortex disorder | Severe visual impairment |
| F4H7100 | Visual cortex disorder due to vascular disorder | Severe visual impairment |
| F4H7300 | Cortical blindness | Severe visual impairment |
| F4H7z00 | Visual cortex disorder NOS | Severe visual impairment |
| F4Hz.00 | Disorder of optic nerve or visual pathway NOS | Severe visual impairment |
| Fy1..00 | Combined visual and hearing impairment | Severe visual impairment |
| Fy1..12 | Deafblind | Severe visual impairment |
| FyuJ.00 | [X]Disorders of optic nerve and visual pathway | Severe visual impairment |
| FyuL.00 | [X]Visual disturbances and blindness | Severe visual impairment |
| FyuL100 | [X]Other visual disturbances | Severe visual impairment |
| SJ0..00 | Optic nerve and pathway injury | Severe visual impairment |
| SJ00.00 | Optic nerve (2nd) injury | Severe visual impairment |
| SJ01.00 | Optic chiasm injury | Severe visual impairment |
| SJ02.00 | Optic pathway injury | Severe visual impairment |
| SJ03.00 | Visual cortex injury | Severe visual impairment |
| SJ0z.00 | Optic nerve or pathway injury NOS | Severe visual impairment |
| SJ0z.11 | Blindness - traumatic - NOS | Severe visual impairment |
| Z96..00 | Provision for visual and hearing impairment | Severe visual impairment |
| Z961.00 | Provision of guide help for visual and hearing impairment | Severe visual impairment |
| Z962.00 | Provision of communicator for visual and hearing impairment | Severe visual impairment |
| Z9E2.00 | Optical low vision aid provision | Severe visual impairment |
| Z9E3.00 | Provision of optical low vision aid - near | Severe visual impairment |
| Z9E3100 | Provision of magnifier low vision aid - near | Severe visual impairment |
| Z9E3200 | Provision of low vision hand magnifier | Severe visual impairment |
| Z9E3300 | Provision of low vision stand magnifier | Severe visual impairment |
| Z9E3500 | Provision of spectacle low vision aid - near | Severe visual impairment |
| Z9E3600 | Provision of telescopic spectacles | Severe visual impairment |
| Z9E3700 | Provision of spectacle magnifier | Severe visual impairment |
| Z9E3900 | Near low vision aid - clip-on spectacle magnifier | Severe visual impairment |
| Z9E3A00 | Provision of spectacle telescope | Severe visual impairment |
| Z9E3B00 | Near low vision aid - integral spectacle telescope | Severe visual impairment |
| Z9E3C00 | Near low vision aid - clip-on spectacle telescope | Severe visual impairment |
| Z9E3D00 | Near low vision aid - extra cap for telescope | Severe visual impairment |
| Z9E3E00 | Provision of headband telescope | Severe visual impairment |
| Z9E4.00 | Provision of optical low vision aid - distance | Severe visual impairment |
| Z9E5.00 | Provision of non-optical low vision aid | Severe visual impairment |
| Z9E5200 | Provision of closed circuit television | Severe visual impairment |
| Z9E5300 | Provision of image intensifier | Severe visual impairment |
| Z9E5400 | Provision of ancillary low vision aid | Severe visual impairment |
| Z9E5700 | Provision of work board | Severe visual impairment |
| Z9E6.00 | Provision of visual appliance | Severe visual impairment |
| Z9E6500 | Provision of audiotaped services | Severe visual impairment |
| Z9E6600 | Provision of talking book | Severe visual impairment |
| ZN56800 | Blind telephone user | Severe visual impairment |
| ZV410 | [V]Problems with sight | Severe visual impairment |
| ZV52200 | [V]Fitting or adjustment of artificial eye | Severe visual impairment |
| 7007300 | Insertion of auditory implant to brainstem | Severe hearing impairment |
| 7308400 | Placement of hearing implant in external ear | Severe hearing impairment |
| 7308500 | Attention to hearing implant in external ear | Severe hearing impairment |
| 7308600 | Removal of hearing implant from external ear | Severe hearing impairment |
| 7311A00 | Insertn bone anchors subcutaneous bone anchored hearing aid | Severe hearing impairment |
| 7317E00 | Removal of hearing implant from middle ear | Severe hearing impairment |
| 7319000 | Insertion fixtures bone anchored hearing prosthesis Stage 1 | Severe hearing impairment |
| 7319100 | Insertion fixtures bone anchored hearing prosthesis Stage 2 | Severe hearing impairment |
| 7319200 | Reduction soft tissue for bone anchored hearing prosthesis | Severe hearing impairment |
| 7319300 | Attention to fixtures for bone anchored hearing prosthesis | Severe hearing impairment |
| 7319400 | One stage insert fixtures bone anchored hearing prosthesis | Severe hearing impairment |
| 7319500 | Fitting external hearing prosthesis bone anchored fixtures | Severe hearing impairment |
| 1C13.00 | Deafness | Severe hearing impairment |
| 1C13300 | Bilateral deafness | Severe hearing impairment |
| 2BL..11 | O/E - deaf | Severe hearing impairment |
| 2BL3.00 | O/E - significantly deaf | Severe hearing impairment |
| 2BL4.00 | O/E - very deaf | Severe hearing impairment |
| 2BL5.00 | O/E - completely deaf | Severe hearing impairment |
| 2DG..00 | Hearing aid worn | Severe hearing impairment |
| 2DH0.00 | Uses hearing loop | Severe hearing impairment |
| 7317C00 | Placement of hearing implant in middle ear | Severe hearing impairment |
| 7317D00 | Attention to hearing implant in middle ear | Severe hearing impairment |
| 7319.00 | Attachment of bone anchored hearing prosthesis | Severe hearing impairment |
| 7319y00 | Other specified attachment bone anchored hearing prosthesis | Severe hearing impairment |
| 7319z00 | Attachment of bone anchored hearing prosthesis NOS | Severe hearing impairment |
| 8D2..00 | Auditory aid | Severe hearing impairment |
| 8D2..11 | Auditory aid provision | Severe hearing impairment |
| 8D2..12 | Hearing aid provision | Severe hearing impairment |
| 8D21.00 | Provide head worn hearing aid | Severe hearing impairment |
| 8D22.00 | Provide body worn hearing aid | Severe hearing impairment |
| 8D23.00 | Ear fitting hearing aid | Severe hearing impairment |
| 8D24.00 | Replace hearing aid battery | Severe hearing impairment |
| 8D25.00 | Physiolog. hearing assistance | Severe hearing impairment |
| 8D2Z.00 | Auditory aid NOS | Severe hearing impairment |
| 8E3..00 | Deafness remedial therapy | Severe hearing impairment |
| 8E3Z.00 | Deafness remedial therapy NOS | Severe hearing impairment |
| 8M41.00 | Hearing aid requested | Severe hearing impairment |
| 9N0b.00 | Seen in hearing aid clinic | Severe hearing impairment |
| 9NfB.00 | Requires deafblind communicator guide | Severe hearing impairment |
| A560200 | Rubella deafness | Severe hearing impairment |
| F581211 | Noise induced deafness | Severe hearing impairment |
| F59..11 | Deafness | Severe hearing impairment |
| F590.11 | Conductive deafness | Severe hearing impairment |
| F591.13 | Perceptive deafness | Severe hearing impairment |
| F591211 | Nerve deafness | Severe hearing impairment |
| F591400 | Congenital sensorineural deafness | Severe hearing impairment |
| F591500 | Ototoxicity - deafness | Severe hearing impairment |
| F591511 | Drug ototoxicity - deafness | Severe hearing impairment |
| F591800 | Congenital prelingual deafness | Severe hearing impairment |
| F592.00 | Mixed conductive and sensorineural deafness | Severe hearing impairment |
| F593.00 | Deaf mutism, NEC | Severe hearing impairment |
| F596.00 | Maternally inherited deafness | Severe hearing impairment |
| F598.00 | Moderate acquired hearing loss | Severe hearing impairment |
| F599.00 | Severe acquired hearing loss | Severe hearing impairment |
| F59A.00 | Profound acquired hearing loss | Severe hearing impairment |
| F59A.11 | Deafened | Severe hearing impairment |
| F59z.00 | Deafness NOS | Severe hearing impairment |
| F59z.11 | Chronic deafness | Severe hearing impairment |
| Fy1..00 | Combined visual and hearing impairment | Severe hearing impairment |
| Fy1..12 | Deafblind | Severe hearing impairment |
| FyuU000 | [X]Deaf mutism, not elsewhere classified | Severe hearing impairment |
| P40z.11 | Deafness due to congenital anomaly NEC | Severe hearing impairment |
| SJ15.12 | Deafness - traumatic - NOS | Severe hearing impairment |
| Z8B5.00 | Ability to use hearing aid | Severe hearing impairment |
| Z8B5100 | Able to use hearing aid | Severe hearing impairment |
| Z8B5200 | Unable to use hearing aid | Severe hearing impairment |
| Z8B5300 | Does use hearing aid | Severe hearing impairment |
| Z8B5311 | Uses hearing aid | Severe hearing impairment |
| Z8B5500 | Difficulty using hearing aid | Severe hearing impairment |
| Z911.00 | Hearing aid procedure | Severe hearing impairment |
| Z911100 | Fit hearing aid | Severe hearing impairment |
| Z911300 | Adjust hearing aid settings | Severe hearing impairment |
| Z911400 | Changing hearing aid battery | Severe hearing impairment |
| Z911500 | Checking hearing aid | Severe hearing impairment |
| Z911700 | Switching on hearing aid | Severe hearing impairment |
| Z911800 | Turning off hearing aid | Severe hearing impairment |
| Z911900 | Putting on hearing aid | Severe hearing impairment |
| ZE87.00 | Hearing loss | Severe hearing impairment |
| ZE87.16 | HL - Hearing loss | Severe hearing impairment |
| Z911A00 | Listening for feedback whistle of hearing aid | Severe hearing impairment |
| Z911B00 | Attention to hearing aid | Severe hearing impairment |
| Z911E00 | Fit ear mould for existing hearing aid | Severe hearing impairment |
| ZE87.00 | Hearing loss Severe Hearing impairment | Severe hearing impairment |
| ZE87.16 | HL - Hearing loss Severe Hearing impairment | Severe hearing impairment |
| Z9E8100 | Hearing aid provision | Severe hearing impairment |
| Z9E8111 | Auditory aid provision | Severe hearing impairment |
| ZE83200 | Hearing for loud voice impaired | Severe hearing impairment |
| ZE84200 | Hearing for voice impaired | Severe hearing impairment |
| ZE87.11 | Deafness | Severe hearing impairment |
| ZE87.13 | Hard of hearing | Severe hearing impairment |
| ZE87.17 | HOH - Hard of hearing | Severe hearing impairment |
| ZN56900 | Deaf telephone user | Severe hearing impairment |
| ZT12711 | Voice associated with hearing loss | Severe hearing impairment |
| ZV45G00 | [V]Presence of external hearing-aid | Severe hearing impairment |
| ZV45N00 | [V]Bone anchored hearing aid in situ | Severe hearing impairment |
| ZV53200 | [V]Fitting or adjustment of hearing aid | Severe hearing impairment |
| ZV53D00 | [V]Adjustment and management of implanted hearing device | Severe hearing impairment |
| 7319600 | First stge ins fixtures for bone anchored hearing prosthesis | Severe hearing impairment |
| 7319700 | Second stage ins fixtures for bone anchored hearing prosth | Severe hearing impairment |
| 13o8M00 | Uses sign language | Severe hearing impairment |
| 13ZL.00 | Using lip-reading | Severe hearing impairment |
| 13ZM.00 | Using British sign language | Severe hearing impairment |
| 13ZP.00 | Using Makaton sign language | Severe hearing impairment |
| 2DH1.00 | Does use hearing aid | Severe hearing impairment |
| 8D26.00 | Provision of replacement hearing aid | Severe hearing impairment |
| 9Nfa.00 | Requires deafblind manual alphabet interpreter | Severe hearing impairment |
| 9Nfb.00 | Requires deafblind block alphabet interpreter | Severe hearing impairment |
| 9NnR.00 | Visual frame sign language interpreter needed | Severe hearing impairment |
| 9NnS.00 | Hands-on signing interpreter needed | Severe hearing impairment |
| F591900 | Bilateral profound sensorineural hearing loss | Severe hearing impairment |
| F591A00 | Bilateral congenital sensorineural hearing loss | Severe hearing impairment |
| F591B00 | Profound sensorineural hearing loss | Severe hearing impairment |
| F591E00 | Severe sensorineural hearing loss | Severe hearing impairment |
| F594.00 | High frequency deafness | Severe hearing impairment |
| F595.00 | Low frequency deafness | Severe hearing impairment |
| Z96..00 | Provision for visual and hearing impairment | Severe hearing impairment |
| Z961.00 | Provision of guide help for visual and hearing impairment | Severe hearing impairment |
| Z962.00 | Provision of communicator for visual and hearing impairment | Severe hearing impairment |
| Z9E3314 | Auditory aid provision | Severe hearing impairment |
| Z9E8.00 | Provision of auditory appliance | Severe hearing impairment |
| ZE83300 | Unable to hear loud voice | Severe hearing impairment |
| ZN56A00 | Deaf-blind telephone user | Severe hearing impairment |
| ZV53011 | [V]Fitting or adjustment of auditory substitution device | Severe hearing impairment |
| 2835.00 | O/E - paraplegia | Severe mobility difficulties |
| 2836.00 | O/E - quadriplegia | Severe mobility difficulties |
| 3960.00 | Dependent: chair/bed transfer | Severe mobility difficulties |
| 3961.00 | Able to sit: chair/bed trans. | Severe mobility difficulties |
| 3980.00 | Immobile | Severe mobility difficulties |
| 3981.00 | Independent in wheelchair | Severe mobility difficulties |
| 3982.00 | Minimal help in wheelchair | Severe mobility difficulties |
| 3990.00 | Unable to climb stairs | Severe mobility difficulties |
| 13C5.00 | Confined to chair | Severe mobility difficulties |
| 13C5.11 | Chairbound | Severe mobility difficulties |
| 13C6.00 | Bed-ridden | Severe mobility difficulties |
| 13C6.11 | Bedbound | Severe mobility difficulties |
| 13CC.00 | Immobile | Severe mobility difficulties |
| 13CD.00 | Mobility very poor | Severe mobility difficulties |
| 13CE.00 | Mobility poor | Severe mobility difficulties |
| 398A.00 | Dependent on helper pushing wheelchair | Severe mobility difficulties |
| 8D9..13 | Wheel chair | Severe mobility difficulties |
| 8D92.00 | Self propelled wheel chair | Severe mobility difficulties |
| 8D93.00 | Pedal powered wheel chair | Severe mobility difficulties |
| 8D94.00 | Powered wheel chair | Severe mobility difficulties |
| 8D95.00 | Wheel chair unspecified | Severe mobility difficulties |
| 8D9A.00 | Attendant powered wheel chair | Severe mobility difficulties |
| 8D9B.00 | Wheel chair seating | Severe mobility difficulties |
| 8HHC.00 | Referred for wheelchair assessment | Severe mobility difficulties |
| 8N1..00 | Modification of wheelchair | Severe mobility difficulties |
| 8T0M.00 | Referral to wheelchair service | Severe mobility difficulties |
| 9R43.00 | Wheelchair in need of repair | Severe mobility difficulties |
| 9R44.00 | Wheelchair in good repair | Severe mobility difficulties |
| 9RA..00 | Wheelchair applied for | Severe mobility difficulties |
| F240.00 | Quadriplegia | Severe mobility difficulties |
| F240.11 | Tetraplegia | Severe mobility difficulties |
| F240100 | Spastic tetraplegia | Severe mobility difficulties |
| F241.00 | Paraplegia | Severe mobility difficulties |
| F241100 | Spastic paraplegia | Severe mobility difficulties |
| F242.00 | Diplegia of upper limbs | Severe mobility difficulties |
| F243.00 | Monoplegia of lower limb | Severe mobility difficulties |
| F244.00 | Monoplegia of upper limb | Severe mobility difficulties |
| N233100 | Immobility syndrome | Severe mobility difficulties |
| R00A.00 | [D]Poor mobility | Severe mobility difficulties |
| R00C.00 | [D]Immobility | Severe mobility difficulties |
| U105.00 | [X]Fall involving wheelchair | Severe mobility difficulties |
| U105000 | [X]Fall involving wheelchair, occurrence at home | Severe mobility difficulties |
| U105100 | [X]Fall involvng wheelchair occurrence residential instit'n | Severe mobility difficulties |
| U105200 | [X]Fall invlv w'chair occ school oth instit/pub admin area | Severe mobility difficulties |
| U105500 | [X]Fall involvng wheelchair occurrnce at trade/service area | Severe mobility difficulties |
| U105700 | [X]Fall involving wheelchair, occurrence on farm | Severe mobility difficulties |
| U105y00 | [X]Fall involv wheelchair, occurrnce at other specif place | Severe mobility difficulties |
| U105z00 | [X]Fall involving wheelchair occurrnce at unspecified place | Severe mobility difficulties |
| Z6R3.00 | Wheelchair dancing therapy | Severe mobility difficulties |
| Z6R8100 | Wheelchair sport | Severe mobility difficulties |
| Z6X1.00 | Wheelchair transfer practice | Severe mobility difficulties |
| Z6Z..00 | Wheelchair education | Severe mobility difficulties |
| Z6Z1.00 | Wheelchair use training | Severe mobility difficulties |
| Z6Z1200 | Propelling wheelchair training | Severe mobility difficulties |
| Z6Z1300 | Controlling electric wheelchair training | Severe mobility difficulties |
| Z9EH400 | Provision of wheelchair | Severe mobility difficulties |
| ZO2..00 | Unable to mobilise | Severe mobility difficulties |
| ZO4..00 | Does not mobilise | Severe mobility difficulties |
| ZO72.00 | Unable to mobilise indoors | Severe mobility difficulties |
| ZO74.00 | Does not mobilise indoors | Severe mobility difficulties |
| ZO75.00 | Difficulty mobilising indoors | Severe mobility difficulties |
| ZO82.00 | Unable to mobilise outside | Severe mobility difficulties |
| ZO84.00 | Does not mobilise outside | Severe mobility difficulties |
| ZO92.00 | Unable to mobilise using mobility aids | Severe mobility difficulties |
| ZO94.00 | Does not mobilise using mobility aids | Severe mobility difficulties |
| ZO96.00 | Ability to mobilise using wheelchair | Severe mobility difficulties |
| ZO96.11 | Wheelchair mobility | Severe mobility difficulties |
| ZO96100 | Able to mobilise using wheelchair | Severe mobility difficulties |
| ZO96200 | Unable to mobilise using wheelchair | Severe mobility difficulties |
| ZO96300 | Does mobilise using wheelchair | Severe mobility difficulties |
| ZO96311 | Mobilises using wheelchair | Severe mobility difficulties |
| ZO96400 | Does not mobilise using wheelchair | Severe mobility difficulties |
| ZO96500 | Difficulty mobilising using wheelchair | Severe mobility difficulties |
| ZOC6200 | Unable to get in and out of a chair | Severe mobility difficulties |
| ZOC6400 | Does not get in and out of a chair | Severe mobility difficulties |
| ZOC8200 | Unable to get out of a chair | Severe mobility difficulties |
| ZOC8400 | Does not get out of a chair | Severe mobility difficulties |
| ZOC9200 | Unable to get on and off a bed | Severe mobility difficulties |
| ZOC9400 | Does not get on and off a bed | Severe mobility difficulties |
| ZOCA200 | Unable to get on a bed | Severe mobility difficulties |
| ZOCB200 | Unable to get off a bed | Severe mobility difficulties |
| ZOCB400 | Does not get off a bed | Severe mobility difficulties |
| ZOD2.00 | Unable to move in bed | Severe mobility difficulties |
| ZOD4.00 | Does not move in bed | Severe mobility difficulties |
| ZOD6200 | Unable to roll over in bed | Severe mobility difficulties |
| ZOD6211 | Unable to turn over in bed | Severe mobility difficulties |
| ZOD7200 | Unable to turn onto side in bed | Severe mobility difficulties |
| ZOD8200 | Unable to move up and down bed | Severe mobility difficulties |
| ZV46200 | [V]Dependence on wheelchair | Severe mobility difficulties |
| ZV4L011 | [V] Poor mobility | Severe mobility difficulties |
| ZV53800 | [V]Fitting or adjustment of wheelchair | Severe mobility difficulties |
| F137.11 | Athetoid cerebral palsy | Cerebral palsy |
| F137.12 | Athetosis - congenital | Cerebral palsy |
| F137000 | Athetoid cerebral palsy | Cerebral palsy |
| F137010 | Vogt's disease | Cerebral palsy |
| F137100 | Double athetosis | Cerebral palsy |
| F137111 | Congenital athetosis | Cerebral palsy |
| F137y00 | Other specified symptomatic torsion dystonia | Cerebral palsy |
| F137z00 | Symptomatic torsion dystonia NOS | Cerebral palsy |
| F23..00 | Congenital cerebral palsy | Cerebral palsy |
| F23..11 | Congenital spastic cerebral palsy | Cerebral palsy |
| F23..12 | Infantile cerebral palsy | Cerebral palsy |
| F23..13 | Littles disease | Cerebral palsy |
| F23..14 | Cerebral atonia | Cerebral palsy |
| F230.00 | Congenital diplegia | Cerebral palsy |
| F230.11 | Paraplegia - congenital | Cerebral palsy |
| F230000 | Congenital paraplegia | Cerebral palsy |
| F230100 | Cerebral palsy with spastic diplegia | Cerebral palsy |
| F230111 | Spastic diplegic cerebral palsy | Cerebral palsy |
| F230z00 | Congenital diplegia NOS | Cerebral palsy |
| F231.00 | Congenital hemiplegia | Cerebral palsy |
| F232.00 | Congenital quadriplegia | Cerebral palsy |
| F232.11 | Tetraplegia - congenital | Cerebral palsy |
| F233.00 | Congenital monoplegia | Cerebral palsy |
| F233.11 | Congenital spastic foot | Cerebral palsy |
| F234.00 | Infantile hemiplegia NOS | Cerebral palsy |
| F23y.00 | Other congenital cerebral palsy | Cerebral palsy |
| F23y000 | Ataxic infantile cerebral palsy | Cerebral palsy |
| F23y100 | Flaccid infantile cerebral palsy | Cerebral palsy |
| F23y200 | Spastic cerebral palsy | Cerebral palsy |
| F23y300 | Dyskinetic cerebral palsy | Cerebral palsy |
| F23y400 | Ataxic diplegic cerebral palsy | Cerebral palsy |
| F23y500 | Worster-Drought syndrome | Cerebral palsy |
| F23y511 | Congenital suprabulbar paresis | Cerebral palsy |
| F23y600 | Choreoathetoid cerebral palsy | Cerebral palsy |
| F23yz00 | Other infantile cerebral palsy NOS | Cerebral palsy |
| F23z.00 | Congenital cerebral palsy NOS | Cerebral palsy |
| F2B..00 | Cerebral palsy | Cerebral palsy |
| F2B0.00 | Spastic quadriplegic cerebral palsy | Cerebral palsy |
| F2B1.00 | Spastic hemiplegic cerebral palsy | Cerebral palsy |
| F2B2.00 | Bilateral spastic cerebral palsy | Cerebral palsy |
| F2By.00 | Other cerebral palsy | Cerebral palsy |
| F2Bz.00 | Cerebral palsy NOS | Cerebral palsy |
| F2Bz.00 | Cerebral palsy NOS | Cerebral palsy |
| Fyu9.00 | [X]Cerebral palsy and other paralytic syndromes | Cerebral palsy |
| Fyu9000 | [X]Other infantile cerebral palsy | Cerebral palsy |
| G669.00 | Cerebral palsy, not congenital or infantile, acute | Cerebral palsy |
| 7615.11 | Gastrojejunostomy, transposed | PEG feeding |
| 7617.00 | Gastrostomy operations | PEG feeding |
| 7617.12 | Creation of gastrostomy | PEG feeding |
| 7619.11 | Gastrotomy NEC | PEG feeding |
| 7632.00 | Jejunostomy | PEG feeding |
| 7607211 | Insertion of feeding tube via artificial oesophageal opening | PEG feeding |
| 7617000 | Creation of permanent gastrostomy | PEG feeding |
| 7617111 | Creation of gastrostomy NEC | PEG feeding |
| 7617200 | Reconstruction of gastrostomy | PEG feeding |
| 7617400 | Attention to gastrostomy tube | PEG feeding |
| 7617500 | Removal of gastrostomy tube | PEG feeding |
| 7617600 | Change of gastrostomy tube | PEG feeding |
| 7617700 | Maintenance of percutaneous endoscopic gastrostomy tube | PEG feeding |
| 7632000 | Creation of jejunostomy | PEG feeding |
| 7632100 | Refashioning of jejunostomy | PEG feeding |
| 7632300 | Creation of feeding jejunostomy | PEG feeding |
| 7822012 | Rodney - Smith hepatojejunostomy+insertion tubal prosthesis | PEG feeding |
| 7617y00 | Other specified gastrostomy operation | PEG feeding |
| 7617z00 | Gastrostomy operation NOS | PEG feeding |
| 761A200 | Open insertion of feeding tube into stomach | PEG feeding |
| 761E400 | Permanent percutaneous endoscopic gastrostomy | PEG feeding |
| 761E600 | Fibreoptic endoscopic percutaneous insert gastrostomy (PEG) | PEG feeding |
| 761E900 | Fibreoptic endoscopic removal of gastrostomy tube | PEG feeding |
| 761EA00 | Fibreoptic endoscopic percutaneous insertion of gastrostomy | PEG feeding |
| 7632y00 | Other specified jejunostomy | PEG feeding |
| 7632z00 | Jejunostomy NOS | PEG feeding |
| 8CJ2.00 | Percutaneous endoscopic gastrostomy feeding | PEG feeding |
| 8CJ3.00 | Jejunostomy feeding | PEG feeding |
| 8CJ4.00 | Button gastrostomy feeding | PEG feeding |
| 8D69.00 | Gastrostomy aid training | PEG feeding |
| 8D6A.00 | Gastrostomy bag fitting | PEG feeding |
| 8D6B.00 | Gastrostomy bag adjustment | PEG feeding |
| 8D6C.00 | Gastrostomy bag changed | PEG feeding |
| J524100 | Complication of gastrostomy | PEG feeding |
| TB03300 | Formation of gastrostomy with complication, without blame | PEG feeding |
| ZC32.54 | PEG - Percutaneous endoscopic gastrostomy feeding | PEG feeding |
| ZC65.00 | Enteral feeding | PEG feeding |
| ZC65100 | Enteral tube feeding | PEG feeding |
| ZC65111 | Tube feeding | PEG feeding |
| ZC65112 | TF - Tube feeding | PEG feeding |
| ZC65200 | Gastrostomy feeding | PEG feeding |
| ZC65300 | Percutaneous endoscopic gastrostomy feeding | PEG feeding |
| ZC65311 | PEG - Percutaneous endoscopic gastrostomy feeding | PEG feeding |
| ZC65400 | Button gastrostomy feeding | PEG feeding |
| ZC65500 | Jejunostomy feeding | PEG feeding |
| ZV44100 | [V]Has gastrostomy | PEG feeding |
| ZV55100 | [V]Attention to gastrostomy | PEG feeding |
| **ICD-10 codes from Hospital Episode statistics to identify severe health needs** | | |
| **ICD-10** | **ICD-10 description** | **Interpretation** |
| G40 | Epilepsy | Epilepsy |
| G41 | Status epilepticus | Epilepsy |
| G06.8 | Epileptic psychosis NOS | Epilepsy |
| F80.3 | Acquired aphasia with epilepsy [Landau-Kleffner] | Epilepsy |
| N39.3 | Stress incontinence | Incontinence |
| N39.4 | Other specified urinary incontinence | Incontinence |
| R15 | Foecal incontinence | Incontinence |
| R32 | Unspecified urinary incontinence | Incontinence |
| F98.0 | Nonorganic enuresis | Incontinence |
| F98.1 | Nonorganic encopresis | Incontinence |
| H54.0 | Blindness, binocular | Severe visual impairment |
| H54.1 | Severe visual impairment, binocular | Severe visual impairment |
| H54.4 | Blindness, monocular | Severe visual impairment |
| H54.5 | Severe visual impairment, monocular | Severe visual impairment |
| H90 | Conductive and sensorinueral hearing loss | Severe hearing impairment |
| H93.0 | Transient ischaemic deafness | Severe hearing impairment |
| H91.0 | Ototoxic hearing loss | Severe hearing impairment |
| H91.2 | Sudden idiopathic hearing loss | Severe hearing impairment |
| H91.3 | Deaf mutism, not elsewhere classified | Severe hearing impairment |
| G82 | Paraplegia and tetraplegia | Severe mobility difficulties |
| G83.1 | Monoplegia of lower limb | Severe mobility difficulties |
| G83.5 | Locked-in syndrome | Severe mobility difficulties |
| Z46.8 | Fitting and adjustment of other specified device - wheelchair | Severe mobility difficulties |
| W05 | Fall involving wheelchair | Severe mobility difficulties |
| Z99.3 | Dependence of wheelchair | Severe mobility difficulties |
| Z74.0 | Need for assistance due to reduced mobility | Severe mobility difficulties |
| R26.3 | Immobility | Severe mobility difficulties |
| G80 | Cerebral palsy | Cerebral palsy |
| Z43.1 | Attention to gastrostomy | PEG feeding |
| Z93.1 | Gastrostomy status | PEG feeding |

**Table S3: Coding of staff role**

| **Role** | **Role description** |
| --- | --- |
| **General practitioner (GP)** | Senior Partner |
|  | Partner |
|  | Assistant |
|  | Associate |
|  | Non-commercial local rota of less than 10 GPS |
|  | Salaried Partner |
|  | Locum |
|  | GP Registrar |
|  | Sole Practitioner |
|  | GP Retainer |
| **Primary care nurse** | Practice Nurse |
| **Allied health/social care professional** | Consultant |
|  | Health Visitor |
|  | Community Nurse |
|  | Midwife |
|  | Community Psychiatric Nurse |
|  | Pharmacist |
|  | Dispenser |
|  | Non-qualified Dispenser |
|  | Physiotherapist |
|  | Chiropodist |
|  | Dietician |
|  | Counsellor |
|  | Other Health Care Professional |
|  | Hospital Nurse |
|  | Community Medical Officer |
|  | School Nurse |
|  | Contact Tracing Nurse |
|  | Stomatherapist |
|  | Acupuncturist |
|  | Mental Handicap Nurse |
|  | Occupational Therapist |
|  | Other Medical & Dental |
|  | Other Nursing & Midwifery |
|  | Other Allied Health Professionals |
|  | Other Healthcare Scientists |
|  | Clinical Practitioner Access Role |
|  | Nurse Access Role |
|  | Nurse Manager Access Role |
|  | Health Professional Access Role |
|  | Dentist |
|  | Osteopath |
|  | Chiropractor |
|  | Homeopath |
|  | Speech Therapist |
|  | Optometrist |
|  | Social Worker |
| **Other** | Commercial Deputising service |
|  | Practice Manager |
|  | Fund Manager |
|  | Business Manager |
|  | Administrator |
|  | Secretary |
|  | Receptionist |
|  | Maintenance staff |
|  | Health Education Officer |
|  | Computer Manager |
|  | Interpreter/Link Worker |
|  | Carer |
|  | Phlebotomist |
|  | Other Students |
|  | Other Professional Scientific & Technical |
|  | Other Additional Clinical Services |
|  | Other Admin & Clerical |
|  | Biomedical Scientist Access Role |
|  | Clinical Coder Access Role |
| **Missing/not known** | Data Not Entered |

**Table S4: Coding for consultation type (any role)**

| **Interpretation** | **Code** | **Description** |
| --- | --- | --- |
| **Face-to-face** | 1 | Clinic |
|  | 2 | Night visit/Deputising service |
|  | 3 | Follow-up/routine visit |
|  | 4 | Night visit, Local rota |
|  | 6 | Night visit, practice |
|  | 7 | Out of hours, Practice |
|  | 8 | Out of hours, Non Practice |
|  | 9 | Surgery consultation |
|  | 11 | Acute visit |
|  | 24 | Children’s home visit |
|  | 27 | Home Visit |
|  | 28 | Hotel Visit |
|  | 30 | Nursing Home Visit |
|  | 31 | Residential Home Visit |
|  | 32 | Twilight Visit |
|  | 34 | Walk-in Centre |
|  | 36 | Co-op Surgery Consultation |
|  | 37 | Co-op Home Visit |
|  | 40 | Community Clinic |
|  | 50 | Night Visit |
| **Telephone** | 10 | Telephone call from a patient |
|  | 21 | Telephone call to a patient |
|  | 35 | Co-op Telephone advice |
|  | 55 | Telephone Consultation |
| **Mail** | 5 | Mail from patient |
|  | 17 | Mail to patient |
| **Referral/Third party** | 12 | Discharge details |
|  | 13 | Letter from Outpatients |
|  | 22 | Third Party Consultation |
|  | 25 | Day Case Report |
|  | 26 | GOS^a^ 18 report |
|  | 29 | NHS Direct Report |
|  | 41 | Community Nursing Note |
|  | 42 | Community Nursing Report |
|  | 45 | Health Visitor Note |
|  | 46 | Health Visitor Report |
|  | 47 | Hospital Inpatient Report |
|  | 49 | Laboratory Request |
|  | 51 | Radiology Request |
|  | 52 | Radiology Result |
|  | 53 | Referral Letter |
|  | 54 | Social Services Report |
|  | 57 | GP to GP communication transaction |
| **Secondary care** | 20 | Casualty Attendance |
|  | 23 | Hospital Admission |
|  | 38 | Minor Injury Service |
| **Administration** | 19 | Administration |
|  | 43 | Data Transferred from other system |
|  | 44 | Health Authority Entry |
|  | 56 | Template Entry |
| **Other** | 14 | Repeat issue |
|  | 15 | Other |
|  | 16 | Results recording |
|  | 18 | Emergency Consultation |
|  | 33 | Triage |
|  | 39 | Medicine Management |
|  | 48 | Initial Post Discharge Review |
|  | 58 | Non-consultation medication data |
|  | 59 | Non-consultation data |
|  | 61 | Extended hours |

**^a^** GOS: General ophthalmic services

**Table S5: Average number of consultations per year by role, intellectual disability status and presence/absence of health needs**

| **Health need** | | **Intellectual disabilities** | | | **No intellectual disabilities** | | |
| --- | --- | --- | --- | --- | --- | --- | --- |
|  |  | N | Consultation rate per year  (95% CI) | | N | Consultation rate per year  (95% CI) | |
| **GP consultations** | | | | | | | |
| **All** | | **7,794** | **4.88** | **(4.84–4.92)** | **176,807** | **3.16** | **(3.15–3.16)** |
| No health needs | | 4,471 | 3.73 | (3.69–3.78) | 161,829 | 2.90 | (2.90–2.91) |
| 1+ health need | | 3,323 | 6.26 | (6.19–6.33) | 14,978 | 5.93 | (5.90–5.96) |
| 2 + health needs | | 1,424 | 7.39 | (7.28–7.50) | 1,465 | 9.49 | (9.36–9.62) |
| Epilepsy | | 1,511 | 7.05 | (6.94–7.15) | 2,205 | 6.10 | (6.02–6.18) |
| Incontinence | | 1253 | 7.28 | (7.15–7.40) | 7826 | 6.39 | (6.35–6.44) |
| Severe visual impairment | | 1242 | 6.19 | (6.08–6.30) | 1279 | 7.40 | (7.28–7.53) |
| Severe hearing impairment | | 618 | 6.32 | (6.16–6.48) | 5845 | 5.72 | (5.68–5.77) |
| Severe mobility difficulties | | 992 | 8.14 | (8.00–8.29) | 1850 | 10.33 | (10.19–10.46) |
| Cerebral palsy | | 678 | 6.26 | (6.11–6.41) | 267 | 4.25 | (4.06–4.45) |
| PEG feeding | | 152 | 8.37 | (8.00–8.77) | 234 | 9.22 | (8.89–9.57) |
| **Practice nurse consultations** | | | | | | | |
| **All** | | **7,794** | **1.35** | **(1.33–1.37)** | **176,807** | **0.94** | **(0.94–0.94)** |
| No health needs | | 4,471 | 1.11 | (1.09–1.14) | 161,829 | 0.88 | (0.88–0.89) |
| 1+ health need |  | 3,323 | 1.64 | (1.61–1.68) | 14,978 | 1.60 | (1.58–1.61) |
| 2 + health needs | | 1,424 | 1.92 | (1.87–1.98) | 1,465 | 2.17 | (2.10–2.23) |
| Epilepsy | | 1,511 | 1.48 | (1.43–1.53) | 2,205 | 1.48 | (1.44–1.52) |
| Incontinence | | 1,253 | 2.02 | (1.96–2.09) | 7,826 | 1.57 | (1.55–1.59) |
| Severe visual impairment | | 1,242 | 1.83 | (1.77–1.89) | 1,279 | 2.20 | (2.13–2.27) |
| Severe hearing impairment | | 618 | 2.11 | (2.02–2.21) | 5,845 | 1.64 | (1.62–1.67) |
| Severe mobility difficulties | | 992 | 1.73 | (1.66–1.80) | 1,850 | 2.43 | (2.36–2.49) |
| Cerebral palsy | | 678 | 1.33 | (1.26–1.40) | 267 | 0.90 | (0.82–1.00) |
| PEG feeding | | 152 | 1.41 | (1.26–1.58) | 234 | 2.62 | (2.44–2.81) |
| **Allied health professional consultations** | | | | | | | |
| **All** | | **7,794** | **0.85** | **(0.83–0.87)** | **176,807** | **0.55** | **(0.54–0.55)** |
| No health needs | | 4,471 | 0.74 | (0.72–0.76) | 161,829 | 0.50 | (0.50–0.51) |
| 1+ health need | | 3,323 | 0.99 | (0.96–1.02) | 14,978 | 1.01 | (1.00–1.02) |
| 2 + health needs | | 1,424 | 1.09 | (1.05–1.13) | 1,465 | 1.40 | (1.35–1.45) |
| Epilepsy |  | 1,511 | 1.06 | (1.02–1.10) | 2,205 | 0.95 | (0.92–0.99) |
| Incontinence |  | 1,253 | 1.16 | (1.11–1.21) | 7,826 | 1.02 | (1.01–1.04) |
| Severe visual impairment | | 1,242 | 1.08 | (1.03–1.12) | 1,279 | 1.37 | (1.32–1.43) |
| Severe hearing impairment | | 618 | 0.98 | (0.92–1.04) | 5,845 | 1.05 | (1.03–1.08) |
| Severe mobility difficulties | | 992 | 1.05 | (1.00–1.10) | 1,850 | 1.54 | (1.49–1.60) |
| Cerebral palsy |  | 678 | 0.81 | (0.76–0.87) | 267 | 0.66 | (0.59–0.74) |
| PEG feeding |  | 152 | 1.10 | (0.90–1.25) | 234 | 1.45 | (1.32–1.59) |
| **Other consultations** | | | | | | | |
| **All** | | **7,794** | **1.22** | **(1.20–1.24)** | **176,807** | **0.69** | **(0.69–0.69)** |
| No health needs | | 4,471 | 0.89 | (0.87–0.92) | 161,829 | 0.63 | (0.62–0.63) |
| 1+ health need | | 3,323 | 1.62 | (1.59–1.66) | 14,978 | 1.35 | (1.34–1.37) |
| 2 + health needs | | 1,424 | 2.04 | (1.98–2.10) | 1,465 | 1.95 | (1.89–2.01) |
| Epilepsy |  | 1,511 | 1.89 | (1.83–1.94) | 2,205 | 1.38 | (1.34–1.42) |
| Incontinence |  | 1,253 | 1.65 | (1.59–1.71) | 7,826 | 1.31 | (1.29–1.33) |
| Severe visual impairment | | 1,242 | 1.66 | (1.61–1.72) | 1,279 | 1.82 | (1.76–1.88) |
| Severe hearing impairment | | 618 | 1.51 | (1.43–1.59) | 5,845 | 1.46 | (1.43–1.48) |
| Severe mobility difficulties | | 992 | 2.48 | (2.40–2.57) | 1,850 | 1.98 | (1.92–2.04) |
| Cerebral palsy |  | 678 | 1.94 | (1.86–2.03) | 267 | 1.25 | (1.15–1.37) |
| PEG feeding |  | 152 | 2.92 | (2.70–3.16) | 234 | 2.07 | (1.92–2.24) |

^a^ standardised by age (10-year-age groups) and gender for people without intellectual disabilities for the specific health need and role

N: number of individuals

Total number of GP consultations: *n*=58,447 (intellectual disabilities); *n*=991,777 (without intellectual disabilities); nurse consultations: *n*=20,037 (intellectual disabilities); *n=*381,119 (without intellectual disabilities); allied health professional consultations: *n*=15,193 (intellectual disabilities); *n*=292,812 (without intellectual disabilities); other professionals: *n*=19,684 (with intellectual disabilities); *n*=338,520 (without intellectual disabilities)

**Table S6: Mean duration of consultations by intellectual disability status and presence/absence of health needs^a^**

| **Health need** | **Intellectual disabilities** | | | | **No intellectual disabilities** | | |
| --- | --- | --- | --- | --- | --- | --- | --- |
|  | N | | Mean (mins) per year  (95% CI) | | N | Mean (mins) per year  (95% CI) | |
| **GP consultations** | | | | | | | |
| **All** | **5,809** | **9.27** | | **(9.12–9.42)** | **119,607** | **9.41** | **(9.38–9.44)** |
| No health needs | 3,084 | 9.39 | | (9.18–9.60) | 107,489 | 9.41 | (9.38–9.44) |
| 1+ health need | 2,725 | 9.13 | | (8.93–9.33) | 12,118 | 9.43 | (9.34–9.52) |
| 2 + health needs | 1,203 | 9.01 | | (8.71–9.31) | 1,240 | 9.28 | (9.00–9.65) |
| Epilepsy | 1,264 | 8.91 | | (8.60–9.22) | 1,758 | 9.39 | (9.13–9.64) |
| Incontinence | 1,046 | 9.76 | | (9.39–10.14) | 6,432 | 9.74 | (9.61–9.87) |
| Severe visual loss | 1,044 | 9.68 | | (9.34–10.02) | 1,056 | 9.58 | (9.24–9.91) |
| Severe hearing impairment | 516 | 9.51 | | (9.04–9.99) | 4,857 | 9.36 | (9.22–9.50) |
| Severe mobility difficulties | 836 | 8.92 | | (8.53–9.31) | 1,571 | 9.52 | (9.19–9.85) |
| Cerebral palsy | 558 | 8.84 | | (8.38–9.31) | 207 | 9.03 | (8.30–9.75) |
| PEG feeding | 131 | 8.13 | | (7.06–9.20) | 196 | 8.65 | (7.99–9.32) |
| **Nurse consultations** | | | | | | | |
| **All** | **3,459** | **10.77** | | **(10.50**–**11.04)** | **74,585** | **10.33** | **(10.28**–**10.38)** |
| No health needs | 1,714 | 10.62 | | (10.24–11.01) | 66,554 | 10.30 | (10.24–10.35) |
| 1+ health need | 1,745 | 10.91 | | (10.53–11.29) | 18,031 | 10.61 | (10.45–10.76) |
| 2 + health needs | 797 | 10.69 | | (10.12–11.27) | 795 | 10.98 | (10.41–11.54) |
| Epilepsy | 774 | 10.42 | | (9.87–10.98) | 1,029 | 10.65 | (10.19–11.11) |
| Incontinence | 677 | 11.15 | | (10.49–11.81) | 4,148 | 10.73 | (10.51–10.95) |
| Severe visual loss | 741 | 12.55 | | (11.88–13.21) | 718 | 10.84 | (10.28–11.39) |
| Severe hearing impairment | 345 | 11.16 | | (10.32–12.00) | 3,277 | 10.48 | (10.24–10.73) |
| Severe mobility difficulties | 511 | 11.01 | | (10.24–11.78) | 899 | 10.08 | (9.57–10.60) |
| Cerebral palsy | 336 | 9.98 | | (9.21–10.74) | 114 | 9.22 | (8.17–10.27) |
| PEG feeding | 70 | 9.42 | | (7.60–11.25) | 112 | 8.99 | (7.51–10.47) |
| **Allied health professional** | | | | | | | |
| **All** | **2,483** | **8.82** | | **(8.53–9.10)** | **45,903** | **8.70** | **(8.63**–**8.76)** |
| No health needs | 1,266 | 9.19 | | (8.76–9.62) | 40,334 | 8.70 | (8.63–8.78) |
| 1+ health need | 1,217 | 8.43 | | (8.05–8.80) | 5,569 | 8.65 | (8.46–8.84) |
| 2 + health needs | 535 | 8.44 | | (7.88–8.99) | 564 | 8.63 | (8.01–9.26) |
| Epilepsy | 598 | 7.99 | | (7.47–8.52) | 705 | 8.43 | (7.91–8.94) |
| Incontinence | 447 | 8.92 | | (8.22–9.62) | 2,802 | 8.62 | (8.33–8.90) |
| Severe visual loss | 482 | 8.91 | | (8.31–9.50) | 520 | 9.10 | (8.44–9.76) |
| Severe hearing impairment | 230 | 9.40 | | (8.47–10.33) | 2,287 | 8.75 | (8.45–9.05) |
| Severe mobility difficulties | 362 | 8.14 | | (7.39–8.88) | 703 | 8.64 | (8.06–9.23) |
| Cerebral palsy | 224 | 7.91 | | (7.05–8.77) | 84 | 7.57 | (6.06–9.09) |
| PEG feeding | 62 | 6.83 | | (5.31–8.36) | 87 | 7.56 | (6.54–8.58) |
| **Other** | | | | | | | |
| **All** | **2,308** | **7.07** | | **(6.65–7.50)** | **42,269** | **5.65** | **(5.58–5.72)** |
| No health needs | 1,091 | 7.71 | | (6.99–8.43) | 37,128 | 5.60 | (5.52–5.68) |
| 1+ health need | 1,217 | 6.50 | | (6.02–6.99) | 5,141 | 6.01 | (5.78–6.23) |
| 2 + health needs | 565 | 6.22 | | (5.54–6.90) | 574 | 6.32 | (5.62–7.03) |
| Epilepsy | 586 | 6.51 | | (5.82–7.19) | 735 | 6.36 | (5.73–6.99) |
| Incontinence | 448 | 6.62 | | (5.81–7.43) | 2,657 | 5.89 | (5.59–6.20) |
| Severe visual loss | 459 | 5.66 | | (4.98–6.34) | 458 | 6.19 | (5.44–6.94) |
| Severe hearing impairment | 222 | 7.67 | | (6.24–9.10) | 2,073 | 5.98 | (5.65–6.30) |
| Severe mobility difficulties | 396 | 6.31 | | (5.55–7.08) | 696 | 6.75 | (6.00–7.49) |
| Cerebral palsy | 242 | 6.54 | | (5.55–7.53) | 79 | 5.58 | (3.72–7.44) |
| PEG feeding | 72 | 5.54 | | (4.24–6.84) | 88 | 6.59 | (4.90–8.28) |

^a^ Individuals with 1+ consultation only

N: number of individuals

Total number of GP consultations: *n*=56,462 (intellectual disabilities); *n*=934,577 (without intellectual disabilities); nurse consultations: *n*=15,702 (intellectual disabilities); *n=*278,897 (without intellectual disabilities); allied health professional consultations: *n*=9,882 (intellectual disabilities); *n*=161,908 (without intellectual disabilities); other professionals: *n*=14,198 (intellectual disabilities); *n*=203,982 (without intellectual disabilities)

**Table S7: Sensitivity analyses on consultation rates (GP only)**

| **Health need** | | **Intellectual disabilities** | | | | **No intellectual disabilities** | | |
| --- | --- | --- | --- | --- | --- | --- | --- | --- |
|  |  | Number of individuals | | Consultation rate per year (95% CI) | | Number of individuals | Consultation rate per year (95% CI) | |
| **Sensitivity analysis 1: Population restricted to individuals in the cohort ≥6 months** | | | | | | | | |
| **All** |  | | **5,953** | **4.85** | **(4.81–4.89)** | **142,500** | **3.14** | **(3.14–3.15)** |
| No health needs | | 3,405 | | 3.69 | (3.64–3.74) | 130,564 | 2.89 | (2.89–2.90) |
| 1+ health need | | 2,548 | | 6.25 | (6.19–6.32) | 11,936 | 5.87 | (5.84–5.90) |
| 2 + health needs | | 1,085 | | 7.41 | (7.29–7.52) | 1,077 | 9.35 | (9.22–9.48) |
| **Sensitivity analysis 2: Population restricted to consultations >0.5 and <60 minutes (or none)** | | | | | | | | |
| **All** | | **7,794** | | **4.28** | **(4.24–4.31)** | **176,807** | **2.81** | **(2.81–2.82)** |
| No health needs | | 4,471 | | 3.28 | (3.23–3.32) | 161,829 | 2.59 | (2.58–2.59) |
| 1+ health need | | 3,323 | | 5.48 | (5.42–5.55) | 14,978 | 5.30 | (5.27–5.33) |
| 2 + health needs | | 1,424 | | 6.44 | (6.33–6.54) | 1,465 | 8.42 | (8.30–8.54) |

**Table S8: Sensitivity analyses on duration of appointments (GP only)**

| **Health need** | | **Intellectual disabilities** | | | | **No intellectual disabilities** | | | |
| --- | --- | --- | --- | --- | --- | --- | --- | --- | --- |
|  |  | Number of individuals | | Mean (minutes)  per year  (95% CI) | | Number of individuals | | Mean (minutes) per year  (95% CI) | |
| **Sensitivity analysis 1: Restricted to individuals in the cohort ≥6 months** | | | | | | | | | |
| **All** |  | **5,128** | **9.23** | | **(9.08–9.38)** | **111,122** | **9.40** | | **(9.37–9.43)** |
| No health needs | | 2,742 | 9.32 | | (9.11–9.54) | 100,250 | 9.39 | | (9.36–9.43) |
| 1+ health need | | 2,386 | 9.12 | | (8.91–9.32) | 10,872 | 9.46 | | (9.37–9.54) |
| 2 + health needs | | 1,045 | 9.02 | | (8.73–9.32) | 1,038 | 9.55 | | (9.26–9.85) |
| **Sensitivity analysis 2: Restricted to individuals with consultations >0.5 and <60 minutes** | | | | | | | | | |
| **All** |  | **5,688** | **9.83** | | **(9.69–9.97)** | **116,199** | **10.17** | | **(10.14–10.19)** |
| No health needs | | 3,007 | 9.98 | | (9.78–10.18) | 104,300 | 10.19 | | (10.16–10.22) |
| 1+ health need | | 2,681 | 9.67 | | (9.47–9.86) | 11,899 | 9.97 | | (9.89–10.06) |
| 2 + health needs | | 1,190 | 9.44 | | (9.16–9.72) | 1,227 | 9.59 | | (9.33–9.85) |
